# Supplementary material for: Managing risk in cancer presentation, detection and referral: a qualitative study of primary care staff views
Source: BMJ Open. 2014 Jun 12;4(6):e004820. doi: 10.1136/bmjopen-2014-004820 (PMC4067858; doi:10.1136/bmjopen-2014-004820)
Supplement: Author's manuscript [file bmjopen-2014-004820.draft_revisions.pdf]

Complexity, continuity & conflict: a qualitative study of primary care staff views of initiatives to promote cancer early detection and referral

Journal:	*BMJ Open*
Manuscript ID:	bmjopen-2014-004820
Article Type:	Research
Date Submitted by the Author:	09-Jan-2014
Complete List of Authors:	Cook, Neil; University of Central Lancashire, School of Medicine and Dentistry<br>Thomson, Gillian; University of Central Lancashire, School of Health<br>Dey, Maria; University of Central Lancashire, School of Medicine and Dentistry
**Primary Subject Heading**:	Qualitative research
Secondary Subject Heading:	Public health, General practice / Family practice
Keywords:	QUALITATIVE RESEARCH, PUBLIC HEALTH, Epidemiology < ONCOLOGY

SCHOLARONE™
Manuscripts

For peer review only

Complexity, continuity & conflict: a qualitative study of primary care staff views of initiatives to promote cancer early detection and referral

Neil Cook, Research Assistant, School of Medicine and Dentistry, University of Central Lancashire, Preston, England*

Gillian Thomson, Senior Research Fellow, School of Health, University of Central Lancashire, Preston, England

Paola Dey, Professor of Public Health Epidemiology, School of Medicine and Dentistry, University of Central Lancashire, Preston, England

*corresponding author: Neil Cook, School of Medicine and Dentistry, Greenbank Building, University of Central Lancashire, Adelphi Street, Preston, England PR1 2HE

Email: NCook2@uclan.ac.uk Phone: 01772 893409

Key Words: primary health care, general practice, neoplasms, qualitative research, early detection of cancer.

Word count: 3760

For peer review only - http://bmjopen.bmj.com/site/about/guidelines.xhtml

ABSTRACT

Background

In the UK, there have been a number of national initiatives to promote earlier detection and prompt referral of patients presenting to primary care with signs and symptoms of cancer.

Objective

To explore the barriers and facilitators, in primary care, to early detection and prompt referral of patients with symptoms suggestive of cancer.

Design

Qualitative, descriptive interview study.

Setting

Six practices in North West England.

Participants

Primary care staff.

Method

A random sample of primary care practices, stratified by level of previous engagement with early detection and referral initiatives, were invited to take part in individual or group-based interviews. Interviews were audio-recorded and transcribed. A thematic network analysis approach was used with independent coding by two researchers and consensual validation of themes.

Results

Thirty-nine participants recruited from six practices took part in individual (n=4) or group interviews (n=5). The global theme to emerge from the interviews was that 'managing risk' in primary care was not well understood by those introducing primary care-based cancer initiatives. Three underpinning organising themes were identified: 'complexity', 'continuity' and 'conflict' which reflected issues such as complex symptoms and care systems, patient-led factors and target focused care.

For peer review only - http://bmjopen.bmj.com/site/about/guidelines.xhtml

For peer review only

Conclusion

To improve the successful implementation of early detection and referral initiatives, practice capacity, complexities related to cancer diagnosis, communication issues associated with the care pathways and conflicting opinions about staff roles need to be addressed.

ARTICLE SUMMARY

Strengths and limitations of this study

- The study is, to the best of our knowledge, the first to investigate the attitudes and opinions of primary care staff towards the implementation of cancer early detection and awareness initiatives.
- Findings revealed a number of issues in primary care related to managing risk.
- The sample consisted of individuals from a range of job roles and practices in rural and urban areas.
- The sample was small and drawn from a single area of the country, which may limit the generalisibility of findings.

For peer review only

For peer review only - http://bmjopen.bmj.com/site/about/guidelines.xhtml

INTRODUCTION

Two decades of concerted effort to expedite access to proven effective cancer treatments in the UK has led to improvements in cancer survival rates.[1] Primary care has a key role in improving cancer survival through earlier detection and referral of patients with cancer symptoms. National referral guidelines for suspected cancer have been published and there is a national system for urgent referral (two-week waiting-time initiative).[2] However, evidence suggests that there is further room for improvement.[3] There is practice variation in the use of the 'two-week' initiative, and some patients are seen several times in primary care before referral.[4-7] A significant proportion of patients also present through emergency routes and have poorer survival.[5, 8]

To further support primary care, resources were developed and/or disseminated by the Department of Health's National Awareness and Early Diagnosis Initiative (NAEDI), established in 2008, and the Royal College of General Practitioners (RCGP), including: audit and significant events analysis tools,[4, 5] general practitioner (GP) level cancer profile data, safety netting recommendations and referral prompts.

To understand whether and how primary care-based cancer initiatives could be more effective, we need to understand the barriers and facilitators to the promotion of earlier consultation, detection and referral at individual (GP) and organisational (primary care practice) level. Key initiatives have mainly focused on GPs, but other members of the primary care team may have key roles: an area which has been largely unexplored. We report the findings of a qualitative study of the views and experiences of a range of primary care staff in one region of England, which at that time was covered by the Lancashire and South Cumbria Cancer Network (LSCCN). In this paper we focus on the experiences of practice staff in implementing cancer detection and referral initiatives and the perceived barriers and facilitators to earlier consultation, detection and referral of those with symptoms suggestive of cancer.

METHODS

This was a qualitative descriptive study utilising both individual and group-based interviews. It aimed to recruit staff in six practices with differing levels of engagement (high, medium and low) with cancer early diagnosis initiatives. Information on the level of engagement was collated via the LSCCN at the end of June 2012.

For peer review only - http://bmjopen.bmj.com/site/about/guidelines.xhtml

High engagers had participated in at least one of the following: RCGP cancer audit, attendance at a course on early diagnosis or face-to-face meetings to discuss GP cancer profiles and action planning. Medium engagers had attended at least one regional meeting about cancer awareness. Low engagers were not known to have engaged in any initiatives.

GP practices in LSSCN were stratified into these three groups and a random sample of 10 practices in each group were sent a letter about the study, followed up with a phone call from the research team after one week. Practices were offered participation in a practice-based group interview, or individual interviews if preferred. The aim was to recruit two practices in each stratum. Due to low uptake, two strata (medium and low engagers) were merged and a random sample of a further 15 practices were sent letters.

In practices agreeing to take part, semi-structured, audio-recorded interviews, attended by two researchers, were undertaken between September and October 2012. All transcribed data were entered into NVivo 10 and analysed using a Thematic Network Analysis approach.[9] This involved an iterative and cyclical process of reading and analysis to identify basic, organising and global themes within the dataset. Analysis was undertaken by two authors (GT and NC) independently on six transcripts initially followed by an in-depth discussion, and consensual validation of key themes. A further cycle of independent and collaborative analysis was then undertaken on a further sub-set of transcripts to ensure rigour and authenticity of the themes generated. All thematic decisions were discussed with the third author (PD). The study was approved by the University of Central Lancashire STEM ethics committee and individual written informed consent was obtained from all participants.

RESULTS

Six practices agreed to participate; three 'high engagers' and three 'low/medium engagers'. Practices were geographically spread across the region in urban and rural areas. Thirty-nine participants took part in group (n=5) or individual (n=4) interviews. Job roles included GP (n=9, 23.1%), receptionist (n=7, 17.9%), nurse (n=6, 15.4%), manager (n=6, 15.4%), secretary (n=5, 12.8%), health care assistant (n=3, 7.7%), medical student (n=2, 5.1%) and phlebotomist (n=1, 2.6%).

Overall, practice staff were aware of the two-week waiting-time initiative. There was also good knowledge about the national 'Be Clear on Cancer' public cancer awareness campaigns, but less awareness about other initiatives specifically targeted at primary care. The key global theme to emerge from the interviews related to 'managing risk' within primary care:

For peer review only - http://bmjopen.bmj.com/site/about/guidelines.xhtml

For peer review only

"It's quite a tricky, nebulous area […]. *The nature of general practice is that we're dealing every day with uncertainty…"* (Interview 6, Participant 3)

A key concern of staff was that policymakers and those implementing cancer initiatives within primary care did not fully understand how risk was managed within primary care. Cancer was only one priority within primary care and there was an abundance of initiatives for a variety of conditions which primary care staff were expected to implement. Three underpinning organising themes (and associated basic themes) of 'complexity', 'continuity' and 'conflict' reflected the tensions and difficulties that primary care face in managing risk whilst dealing with complex symptoms and care systems, patient-led factors and target-focused care. An overview of the organising and basic themes is presented in figure 1. These themes are described and discussed, contextualised by participant quotes below.

Complexity

Cancer disease and symptoms: Cancer diagnosis was considered an important priority. However, whilst some symptoms were straightforwardly associated with cancer, e.g. breast lump, other symptoms commonly presenting in practice, compared with the relative infrequency of cancer in a practice population, provided greater challenges:

"How often do we see them […] *most cancers you, sort of, see so infrequently, that it's actually quite difficult to then keep up-to-date with, you know, what you're supposed to be doing and the referral pathways and all that sort of thing, when you might see one or two a year."* (Interview 4, Participant 1)

Fragmentation and access to diagnostic services: Primary care staff expressed difficulties accessing diagnostic services due to fragmented systems and multiple agencies. Rigid referral criteria led to cases being '*bounced back*' if they failed to meet these criteria. However, concerns about patients led many participants to '*fudge*', '*embellish*' or '*bend-the-rules*' to ensure assessment:

"I think, sometimes […] *even though it says on the form, don't fill out this form unless they tick any boxes but you find a box to tick, and usually for very good reason. And I think you'd only go, slightly over egging the presentation if you were pretty sure there was something there, something going on."* (Interview 4, Participant 4)

For peer review only - http://bmjopen.bmj.com/site/about/guidelines.xhtml

Several participants felt frustrated by restricted access to diagnostics for certain conditions:

> *"I can send people for a CT scan if I have a concern about them, for some conditions, but not others. Well why not? I'm the one who's initiating the referral in the first place. You trust me to initiate the referral to pick the patient and prepare them so that you can come along and just arrange the scan and look at it."* (Interview 4, Participant 3)

Access restrictions were felt to reflect *'empire building'* and *'preservation'*, rather than a '*logical'* service delivery model. Whilst some GPs wanted more direct access to diagnostics, this was not universal, and others highlighted the need for appropriate *'training'*.

Guidelines content and information overload: Participants referred to the usefulness of guidelines to help symptom differentiation. Knowledge was felt to be experientially derived; referral guidelines were considered useful for less experienced doctors. Guidance that expressed the statistical probabilities that someone had cancer was less preferred to guidance on what symptoms or signs raise concern or require further investigation:

> *"I mean, you see, from a GPs point of view, if somebody comes in who is fifty-five years of age, and passes blood in his stool, you need to exclude a cancer. Now, I don't want to know how many, percentage of those people pass blood in the motion will have cancer, or how many will have piles or, inflammatory bowel disease or what have you* […]. *Text books don't help you there, NICE guidelines do not help you in that sort of situation."* (Interview 4, Participant 3)

Concerns were expressed about the number of guidelines available for different conditions and inconsistencies between different sources:

> *"And that's the problem 'cause last year there was a big campaign, "if you have a cough for more than three weeks you need to go and see a doctor", but NICE guidelines is six weeks,* […] *so there is no uniformity."* (Interview 6, Participant 4)

Participants described a *'tsunami'* of new guidelines, care plans and initiatives inducing guideline '*negativity'* and difficulties keeping up-to-date:

For peer review only - http://bmjopen.bmj.com/site/about/guidelines.xhtml

> *"And I thought that's probably a squamous cell carcinoma. That needs a two-week wait referral. Then, I actually checked the guidelines, the guidelines say that "it's got to be over one centimetre and you've got to wait more than eight weeks really for it to grow" etcetera, etcetera* […] *and those things will change on a regular basis. Now, how do you expect an entire network of primary care physicians to stay current with all those guidelines and apply them religiously?"* (Interview 2, Participant 3).

Continuity

Patient-practice relationships: Continuity-of-care could not always be achieved in practice, even though patients preferred it and its absence could contribute to diagnostic delay:

> *"People have been hopping around from one clinician to another and that, and you* [...] *don't see the evolution of the story until* […] *it hits you in the face* […]. *Patients book on the day, they don't necessarily get in the person they normally see, they get in with the available, so that can disjoint things…."* (Interview 3, Participant 4)

It was felt that smaller, rural practices had closer relationships with patients and their community, which would lead to earlier consultation. However, others felt awareness of cancer deaths was heightened in close-knit communities, which could reinforce negative views.

Primary care and secondary care interface: Expedited assessment following GP referral of patients through the two-week wait initiative was perceived to work well, but delays following patients' initial appointment were reported. Some staff worried about the impact on patients:

> *"And, well, it's very difficult from our point of view, 'cause we're saying, "look, you know this may be nothing serious, however, I want to refer you on the system, you'll get an appointment in two weeks", and they do get seen, but then there's a massive delay, so, you know, then they're kinda all heightened up because they're thinking, "{whispered} the doctor thinks I've got cancer" and then nothing happens for ages, and it's really hard for the patients…"* (Interview 3, Participant 5)

Delays in hospital communication were also identified:

> *"You know, a letter takes, takes two or three days or four days to be produced, then you have*

For peer review only - http://bmjopen.bmj.com/site/about/guidelines.xhtml

> *to wait a fortnight for a consultant to sign it, because they're away, then it has to go in the internal post to the next department, then it has to go into the system. I mean you just wait, I mean you just see waits stretch ahead."* (Interview 2, Participant 3)

There were concerns that centralising services negatively affected assessment uptake among low income patients. In some rural locations, transport was provided, with financial implications:

> *"I mean, up here, if you haven't got your own transport, you can use the transport service, that again is a cost to the National Health, how much is that costing?"* (Interview 2, Participant 2)

Practice-based follow-up: Practices were divided on whether they should follow patients up if they failed to attend for cancer screening; the debate centred on whether the practice or the patient was responsible:

> *"When we got these figures out we did bash that around quite a bit*[…]*, and we felt that patients had had quite a lot of letters, quite a lot of information, and it would, certainly for the bowel screening and the breast screening, we felt that they actually made their own decisions."* (Interview 3, Participant 4)

> *"Yeah, you can bet your bottom dollar that one of those people who doesn't turn up or refuses to have them done,* […] *statistically one of these people, over the following three years is going to develop a bowel cancer, and if they suddenly discover that there was the screening and the doctor was aware that he didn't turn up, he's gonna take legal action, because he'll say the doctor must have an obligation to remind me."* (Interview 4, Participant 3)

Conflict

Prevention versus target driven care: Cancer was considered a public health priority but many participants considered that there was a tension between preventative approaches and achieving Quality and Outcomes Framework (QOF) targets:

For peer review only - http://bmjopen.bmj.com/site/about/guidelines.xhtml

> *"There is a real public health disconnect in primary care, how we're funded, what we do, and we're not as good as with public health, with local public health initiatives, as maybe we should be necessarily* […]. *It's just time to do things,* […] *and those things aren't required of our core business, and when you have a very detailed contract that tells you what you will be paid for doing…"* (Interview 2, Participant 3)

The routine review of patients with long-term conditions had major resource implications for primary care, particularly small practices. However, others felt that this could present an opportunity:

> *"You've got the long-term condition nurses who are going into a different clientele to those from the district nurses…"* (Interview 2, Participant 2)

Even when QOF focussed on public health issues, there were concerns about the focus on target attainment. For example, GPs repeatedly asking patients opportunistically if they have attended screening led to concerns that this may '*put patients off*' coming to see them because they become '*fed up*' with being asked. Participants also felt they needed to be careful about pushing patients '*too hard*' in case they raised anxiety because they then believed the doctor '*knows something*'.

Staff roles: There were conflicting views around the role of other practice staff in cancer awareness, in particular with regard to reception staff. Some staff viewed the reception role as a health advisor, whereas others perceived their role to be purely administrative. Additionally, there were reservations about the ability of reception staff to provide these messages and how the public might feel about receiving health information from a member of reception staff:

> *"You're passing clinical responsibility to people who are non-clinical. It's alright for patients to speak to them, and that's fine, but, I'm not gonna use the receptionists as a source of information* […]. *I think anything clinical should be passed to the doctor full stop. I don't think there is any other role for the receptionist."* (Interview 4, Participant 3)

There were also concerns about potential litigation issues if reception staff were to give advice and how this could impact on the practice:

> *"I think it's also worth mentioning that at this point, because of our roles, how they are, we get a lot of patients not happy about us supplying information, because we're not allowed to*

For peer review only - http://bmjopen.bmj.com/site/about/guidelines.xhtml

diagnose, obviously for obvious reasons, but if you was to imply something, that could come back on us twice as hard because you'd implied something that could be wrong, and therefore it is now your fault." (Interview 1, Participant 1)

A further issue associated with staff roles was the identified benefits of having a chain of communication between all practice members. For example, receptionists being able to *'raise issues'* with the doctor if they know someone is coming in who has symptoms the doctor should be aware of but the patient *'may not say anything'.* This can also work the other way:

"Patients that I worry about, I will leave messages. I'll mention to reception or mention to people that I want to follow them up." (Interview 6, Participant 3)

Some could see a role for other members of the wider practice team, particularly to support screening uptake:

"We'd say well who are the district nurses already going out to? You know, maybe they carry packs with them [those] *they're regularly going into and it's given by word of mouth. ….you know, you can work with your local pharmacists."* (Interview 2, Participant 2)

Cancer awareness: Although cancer fear was commonly acknowledged, staff were divided as to whether patients were afraid to consult the GP with cancer symptoms. Some felt that unhealthy lifestyle choices contributed to patient reluctance to present with cancer symptoms:

"The cancers where they feel that they may have contributed to it, like smoking, they tend to ignore because they don't want to be told that it may be their fault in a certain way, and they don't want to give up the lifestyle." (Interview 6, Participant 4)

Cancer awareness campaigns were felt to be important, but those who presented to the practice following the campaign were more likely to be those at least risk:

"It's the people who never come to the doctors that you want to hit, not the people that were already coming in anyway, and they tend to be ones that see those adverts. But then if you can get one person who wouldn't normally come in and you catch them, then... it's better." (Interview 5, Participant 1)

For peer review only - http://bmjopen.bmj.com/site/about/guidelines.xhtml

Campaigns were also felt to create extra work for all practice members. There were complaints that cancer awareness campaigns tend to run without consideration of other local or national campaigns which may be running simultaneously:

> *"It's like it's alright saying putting in place to get them in, but it's the sheer* volume*, isn't it? You can only cope with so much can't you?"* (Interview 5, Participant 1)

DISCUSSION

To the best of our knowledge, this is the first study which has qualitatively explored the attitudes and opinions of a range of primary care staff about early consultation, detection and referral initiatives to reduce cancer diagnostic delay. The core theme identified by the study was that of the tensions and difficulties in managing risk in primary care.

The study was small, descriptive and exploratory. However, it covered a diverse geographical area and practices with varying levels of engagement with awareness and early detection initiatives. Furthermore, although exploratory, the findings highlight key issues for cancer diagnosis in primary care, which warrant further investigation and consideration and which can be used to develop theoretical perspectives for larger studies.

Diagnosing cancer in primary care is difficult. Diagnostic errors in cancer are frequent and primary care malpractice claims common.[10] To support practitioners, national referral guidelines have been published.[2] There is limited evidence to suggest that these have, in part, contributed to diagnostic delay reduction in the UK.[3] There is also increasing interest in risk assessment tools, to aid decision-making, with algorithms and probabilities of risk based on demographic characteristics, lifestyle factors, symptoms and/or attendance frequency.[11] Some have been disseminated nationally, with limited evidence of effectiveness.[12] A number of practitioners in our study felt these aids introduced rules which failed to encapsulate practitioners' tacit concerns about patients. Previous work in primary care decision-making emphasised the analytical over intuition, but recent work, including in cancer diagnosis, suggests the experiential knowledge may have a role and may be more responsive to individual patients.[13-16] Further research into general practitioner's diagnostic reasoning in cancer is warranted with particular emphasis on the relationship between analytical and tacit approaches and the implications for further development of risk assessment tools in routine practice.

For peer review only - http://bmjopen.bmj.com/site/about/guidelines.xhtml

Practice staff highlighted that patient-doctor relationships were increasingly being eroded, particularly in large practices. Others suggest that fragmentation of primary care and shorter consultation times has led to a lack of continuity-of-care, which may hinder early diagnosis as cancer presentation can be complex in those with multiple co-morbidities.[4, 17, 18] A recent analysis of significant event audits in lung cancer highlighted how cancer can mimic other diagnoses and have atypical presentation.[4] Other practice team members may be in contact with the patients more regularly. Nurses undertaking regular review of patients with long-term conditions could raise symptom awareness or be more alert to changes in, or new, symptoms suggestive of cancer. Receptionists could act as navigators directing patients with symptoms to see the GP.[19] Low levels of knowledge about some cancer symptoms has been demonstrated in non-medical staff and training needs should be addressed.[20] However, this study highlighted that professional boundaries and concerns about litigation could impede such whole-system working. Research within primary care suggests that facilitation of communities of practice and interdisciplinary knowledge sharing may reduce some of these barriers.[21]

Our findings also suggest that the primary/secondary care interface needs strengthening; an observation supported by others who highlighted the role of poor communication between primary and secondary care in diagnostic delay.[4] While there was almost universal support for the two-week waiting-time initiative, concerns were expressed about patient delays after their first assessment and the time taken for patient information to be sent back to the practice. Practice staff felt it was difficult to support patients during this time and that they could be lost to the system, particularly when hospital services were fragmented. For some, increasing direct access to diagnostic services from primary care was seen as a key mechanism for reducing some of these delays and supporting early referral, but the need for training in referral criteria was also highlighted.

For there to be greater success, initiatives aimed at primary care need to take into account GP practice workload and organisation. A 'one-size-fits-all' approach is unlikely to succeed as practitioners expressed a range of views and opinions on the benefits of different initiatives. Practices vary in size, population demography, community interactions, organisation and staff expectations, which can affect their capacity to enact different initiatives and/or whether their patients can access services. Lack of implementation of initiatives in primary care is not always because of resistance to the initiatives themselves, but sometimes because of the sheer number of initiatives across a range of priority areas that practices are expected to implement, often simultaneously. Policymakers should consider more carefully how new initiatives in cancer early

For peer review only - http://bmjopen.bmj.com/site/about/guidelines.xhtml

detection impact on primary care, how these can be embedded into practice systems and emphasise and exploit synergies with other disease conditions. On the other hand, they also need to consider the impact of primary care policies, designed to benefit other patient groups, on the earlier consultation and referral of patients with signs and symptoms of cancer. A greater appreciation and understanding of these issues by commissioners and policymakers could lead to greater gains in cancer survival.

ACKNOWLEDGEMENTS: Our thanks to the practices who participated in this study and to the staff of the Lancashire and South Cumbria Cancer Network who assisted us.

COMPETING INTERESTS: PD was public health lead for LSCCN and NC is part-funded by a grant from LSCCN.

FUNDING: This work was supported by Lancashire and South Cumbria Cancer Network (LSCCN) as part of the National Cancer Awareness and Early Detection initiative (NAEDI) funding 'cancer networks supporting primary care'.

CONTRIBUTIONS:

NC recruited participants, carried out interviews, transcribed the interviews, coded the transcripts, contributed to the development of the coding framework, and contributed to the writing of the manuscript.

GT carried out interviews, coded the transcripts, contributed to the development of the coding framework and contributed to the writing of the manuscript .

PD designed the study, collated practice engagement data, contributed to the development of the coding of the framework and contributed to the writing of the manuscript.

DATA SHARING: No further data are available

REFERENCES

1. Coleman MP, Foreman D, Bryant H, et al. Cancer survival in Australia, Canada, Denmark, Norway, Sweden, and the UK, 1995—2007 (the International Cancer Benchmarking Partnership): an analysis of population-based cancer registry data. *The Lancet*. 2011;**377**:127–38. doi:10.1016/S0140-6736(10)62231-3.

2. National Institute for Clinical Excellence. Clinical Guideline 27. Referral guidelines for suspected cancer. 2005. http://www.nice.org.uk/nicemedia/live/10968/29814/29814.pdf accessed 3/1/2014.

3. Neal RD, Din NU, Hamilton W, et al. Comparison of cancer diagnostic intervals before and after implementation of NICE guidelines: analysis of data from the UK General Practice Research Database. *Br J Cancer*. 2013. Published online first: Dec 24. doi:10.1038/bjc.2013.791.

For peer review only - http://bmjopen.bmj.com/site/about/guidelines.xhtml

4. Mitchell ED, Rubin G, McLeod U. Understanding diagnosis of lung cancer in primary care: qualitative synthesis of significant event audit reports. *Br J Gen Pract*. 2013;**63**(606):e37-46. doi: 10.3399/bjgp13X660760.

5. Royal College of General Practitioners. Clinical Innovation and Research Centre. National Audit of Cancer Diagnosis in Primary Care. 2011. http://www.rcgp.org.uk/news/2011/november/~/media/Files/News/National_Audit_of_Cancer_Diagnosis_in_Primary-Care.ashx. last accessed 03/01/2014.

6. Lyratzopoulos G, Neal RD, Barbiere JM, et al. Variation in number of general practitioner consultations before hospital referral for cancer: findings from the 2010 National Cancer Patient Experience Survey in England. *Lancet Oncol*. 2012;**13**(4):353-65. doi:10.1016/S1470-2045(12)70041-4.

7. Meechan D, Gildea C, Hollingworth L, et al. Variation in use of the 2-week referral pathway for suspected cancer: A cross-sectional analysis. *Br J Gen Pract*. 2012; 62(602): e590–97. doi:10.3399/bjgp12X654551.

8. Elliss-Brookes L, McPhail S, Ives A, et al. Routes to diagnosis for cancer - determining the patient journey using multiple routine data sets. *Br J Cancer*. 2012;**107**(8):1220-26. doi:10.1038/bjc.2012.408.

9. Attride-Stirling J. Thematic networks: an analytic tool for qualitative research. *Qualitative Research*. 2001:**1**(3):385-405. doi:10.1177/146879410100100307.

10. Wallace E, Lowry J, Smith SM, et al.. The epidemiology of malpractice claims in primary care: a systematic review. *BMJ Open*. 2013;**3**:e002929. doi:10.1136/bmjopen-2013-002929.

11. Emery J. Editorial: Assessment of cancer risk in men and women. *Br J Gen Pract*. 2013;**63**(606):4-5. doi: 10.3399/bjgp13X660607. doi:10.3399/bjgp13X660607.

12. Hamilton W, Green T, Martins T, et al. Evaluation of risk assessment tools for suspected cancer in general practice: a cohort study. *Br J Gen Pract*. 2013;**63**(606):e30-36. doi: 10.3399/bjgp13X660751.

13. Johansen ML, Holtedahl KA, Rudebeck CE. How does the thought of cancer arise in a general practice consultation? Interviews with GPs. *Scand J Prim Health Care*. 2012;**30**(3):135-40. doi:10.3109/02813432.2012.688701.

14. André M, Andén A, Borgquist L, et al. GPs' decision-making-perceiving the patient as a person or a disease. *BMC Fam Pract*. 2012;**13**:38. doi:10.1186/1471-2296-13-38.

15. Stolper E, Van de Wiel M, Van Royen P, et al. Gut feelings as a third track in general practitioners' diagnostic reasoning. *J Gen Intern Med*. 2011;**26**(2):197-203. doi:10.1007/s11606-010-1524-5.

16. Zwolsman S, te Pas E, Hooft L, et al. Barriers to GPs' use of evidence-based medicine: a systematic review. *Br J Gen Pract*. 2012;**62**(600):e511-21. doi: 10.3399/bjgp12X652382.

17. Brindle L, Pope C, Corner J, et al. Eliciting symptoms interpreted as normal by patients with early lung cancer. *BMJ Open*. 2012;**2**:e001977. doi:10.1136/bmjopen-2012-001977.

For peer review only - http://bmjopen.bmj.com/site/about/guidelines.xhtml

18. Round T, Steed L, Shankleman J et al. Primary care delays in diagnosing cancer: what is causing them and what can we do about them? *J R Soc Med*. 2013;**106**(11):437-40. doi:10.1177/0141076813504744.

19. Dohan D,Schrag D. Using navigators to improve care of underserved patients. *Cancer*. 2005;**104**:848-55. doi:10.1002/cncr.21214.

20. Cook N, Hart A, Nuttall K, et al. A telephone survey of cancer awareness among frontline staff: informing training needs. *Br J Cancer*. 2011;**105**(3):340-45. doi:10.1038/bjc.

21. Kislov R, Walshe K, Harvey G. Managing boundaries in primary care service improvement: a developmental approach to communities of practice. *Implement Sci*. 2012;**7**:97. doi:10.1186/1748-5908-7-97.

For peer review only

Figure 1: organising themes of complexity, continuity and conflict with their associated basic themes, centred around the global theme of managing risk in primary care

For peer review only

For peer review only - http://bmjopen.bmj.com/site/about/guidelines.xhtml

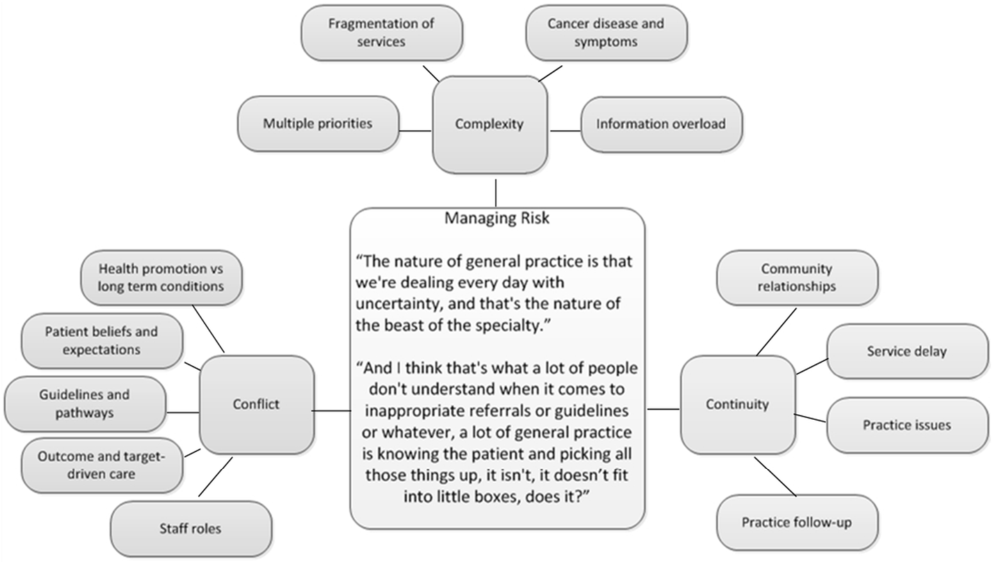

Figure 1: organising themes of complexity, continuity and conflict with their associated basic themes, centred around the global theme of managing risk in primary care

97x54mm (300 x 300 DPI)

review only

BMJ Open

Managing risk in cancer presentation, detection and referral: a qualitative study of primary care staff views

Journal:	*BMJ Open*
Manuscript ID:	bmjopen-2014-004820.R1
Article Type:	Research
Date Submitted by the Author:	16-Apr-2014
Complete List of Authors:	Cook, Neil; University of Central Lancashire, School of Medicine and Dentistry<br>Thomson, Gillian; University of Central Lancashire, School of Health<br>Dey, Maria; University of Central Lancashire, School of Medicine and Dentistry
**Primary Subject Heading**:	Qualitative research
Secondary Subject Heading:	Public health, General practice / Family practice
Keywords:	QUALITATIVE RESEARCH, PUBLIC HEALTH, Epidemiology < ONCOLOGY

SCHOLARONE™
Manuscripts

Managing risk in cancer presentation, detection and referral: a qualitative study of primary care staff views

Neil Cook, Research Assistant, School of Medicine and Dentistry, University of Central Lancashire, Preston, England*

Gillian Thomson, Senior Research Fellow, School of Health, University of Central Lancashire, Preston, England

Paola Dey, Professor of Public Health Epidemiology, School of Medicine and Dentistry, University of Central Lancashire, Preston, England

*corresponding author: Neil Cook, School of Medicine and Dentistry, Greenbank Building, University of Central Lancashire, Adelphi Street, Preston, England PR1 2HE

Email: NCook2@uclan.ac.uk Phone: 01772 893409

Key Words: primary health care, general practice, neoplasms, qualitative research, early detection of cancer.

Word count: abstract- 232, manuscript - 4,504

For peer review only - http://bmjopen.bmj.com/site/about/guidelines.xhtml

ABSTRACT

Objectives: In the UK, there have been a number of national initiatives to promote earlier detection and prompt referral of patients presenting to primary care with signs and symptoms of cancer. The aim of the study was to explore the experiences of a range of primary care staff in promoting earlier presentation, detection and referral of patients with symptoms suggestive of cancer.

Setting: Six primary care practices in North West England.

Participants: Thirty-nine primary care staff from a variety of disciplines took part in five group and four individual interviews.

Results: The global theme to emerge from the interviews was 'managing risk', which had three underpinning organising themes: 'complexity', relating to uncertainty of cancer diagnoses, service fragmentation and plethora of guidelines; 'continuity', relating to relationships between practice staff and their patients and between primary and secondary care; 'conflict' relating to policy drivers and staff role boundaries. A key concern of staff was that policymakers and those implementing cancer initiatives did not fully understand how risk was managed within primary care.

Conclusion: Primary care staff expressed a range of views and opinions on the benefits of cancer initiatives. National initiatives did not appear to wholly resolve issues in managing risk for all practitioners. Staff were concerned about the number of guidelines and priorities

For peer review only

they were expected to implement. These issues need to be considered by policymakers when developing and implementing new initiatives.

ARTICLE SUMMARY

Strengths and limitations of this study

- The study investigates the experiences of a range of primary care staff around implementing initiatives for the earlier diagnosis of cancer.
- The underlying concern in primary care is related to managing risk.
- The sample included a mix of practices with different practice characteristics and included those known to be engaged in national and regional initiatives and those who were less engaged.
- The sample was drawn from a single English region.

For peer review only

For peer review only - http://bmjopen.bmj.com/site/about/guidelines.xhtml

INTRODUCTION

Studies in the 1990s showed that UK cancer survival rates were worse than many other European countries, following which there has been two decades of concerted effort to expedite access to proven effective cancer treatments.[1] While there have been improvements in cancer survival latterly, the UK still lags behind many other countries with similar health care systems, which may be partly due to later stage of disease at presentation.[2]

Both nationally and internationally, primary care is seen to have a key role in improving cancer survival by reducing delays in diagnosis through promoting earlier presentation and through earlier detection and referral of those with symptoms for further specialist assessment.[3, 4] In the UK, national campaigns extol those with symptoms to see their general practitioner earlier; there are national referral guidelines for suspected cancer and a national system for urgent referral from primary to secondary care (two-week waiting-time initiative).[5] However, evidence suggests that there may be further room for improvement:[6] there is practice variation in the use of the 'two-week' initiative and some patients are seen several times in primary care before referral.[7-9] A significant proportion of patients also present through emergency routes and have poorer survival.[10]

To further support primary care in the UK, resources were developed and/or disseminated by the Department of Health's National Awareness and Early Diagnosis Initiative (NAEDI), established in 2008, and the Royal College of General Practitioners (RCGP) including: audit and significant events analysis tools,[7, 8] general practitioner (GP) level cancer profile data, safety netting recommendations and risk assessment tools.[11] Key initiatives have mainly

For peer review only - http://bmjopen.bmj.com/site/about/guidelines.xhtml

focused on GPs, but other members of the primary care team may have key roles: an area which has been largely unexplored.

A number of international studies have been undertaken in attempts to understand the reasons behind delay in cancer diagnosis. Qualitative studies have mostly focussed on patient perspectives.[12-14] Only a few have explored primary care experiences and these have been mainly limited to decision-making processes or referral pathways [11, 15, 16] and from the perspective of the general practitioner. [3, 11, 15,16] In order to understand how a range of initiatives across the patient pathway in primary care could be more effective and the role of other members of the practice team, we explored the experiences of a range of primary care staff in supporting earlier presentation, detection and referral of those with symptoms suggestive of cancer. The study was undertaken in one region of England, which at that time was covered by the Lancashire and South Cumbria Cancer Network (LSCCN).

METHODS

This was a qualitative descriptive study utilising both individual and group-based interviews. It aimed to recruit staff from six practices who were differentially engaged with the national awareness and early diagnosis of cancer agenda. GP practices within LSSCN were stratified into one of three groups at the end of June 2012. High engagers had participated in at least one of the following: RCGP cancer audit, attendance at a course on early diagnosis or face-to-face meetings to discuss GP cancer profiles and action planning. Medium engagers had attended at least one regional meeting about cancer awareness. Low engagers were not

For peer review only - http://bmjopen.bmj.com/site/about/guidelines.xhtml

known to have engaged in any initiatives. Of 254 practices in the geographical area, there were 51 high engagers, 69 medium engagers and 134 low engagers.

Within each group, a random sample of 10 practices were sent a letter about the study, followed up with a phone call from the research team one week later. The initial aim was to recruit at least two practices in each stratum. Due to low uptake within the medium and low engager strata, these categories were merged and a random sample of a further 15 practices sent letters. Practices were offered a choice of either group or individual interviews as it was recognised that time constraints prevent some practice staff from taking part in group interviews and some may feel uncomfortable discussing the issues with colleagues. Individual interviews were the preferred method in only one practice. Interviews (group and individual) were held on only one occasion in each practice.

Six practices agreed to take part in semi-structured, audio-recorded interviews, which were attended by two researchers, between September and October 2012. We recruited three practices in the high stratum and three practices in the merged medium and low stratum (a mix of low and medium engagers). The topic guide is outlined in Box 1. Thirty-nine participants took part in group (n=5) or individual (n=4) interviews. Job roles included GP (n=9), receptionist (n=7), nurse (n=6), manager (n=6), secretary (n=5), health care assistant (n=3), medical student (n=2) and phlebotomist (n=1). Practice characteristics are shown in table 1.

For peer review only - http://bmjopen.bmj.com/site/about/guidelines.xhtml

Table 1: Practice and interview characteristics

Practice characteristic	No. of practices
No of partners	
4 or more	3
3 or less	3
Deprivation Quintile*	
5 or 4 (more deprived)	3
2 or 3	3
1 (most affluent)	0
Population Density**	
<15%	2
15 to 39%	2
>=40%	2

*based on index of multiple deprivation of practice location 2010 (source: Department for Communities and Local Government, Indices of Deprivation 2010)
** Person per hectare based on practice location (source: ONS Neighbourhood Statistics)

The interviews took between 38 and 67 minutes to complete, and between four and eleven staff took part in each practice. All transcribed data were entered into NVivo 10 and analysed using a Thematic Network Analysis approach.[17] This involved an iterative and cyclical process of reading and analysis to identify basic, organising and global themes within the dataset. Analysis was undertaken by two authors (GT and NC) independently on six transcripts initially, followed by an in-depth discussion and consensual validation of key themes. A further cycle of independent and collaborative analysis was then undertaken on a further sub-set of transcripts to ensure rigour and authenticity of the themes generated. All thematic decisions were discussed with the third author (PD). The study was approved by the University of Central Lancashire 'STEMH' ethics committee and individual written informed consent was obtained from all participants.

For peer review only - http://bmjopen.bmj.com/site/about/guidelines.xhtml

RESULTS

Overall, practice staff were well aware of the two-week waiting-time initiative and had good knowledge about the national 'Be Clear on Cancer' cancer awareness public campaigns, but had less awareness about other initiatives specifically targeted at primary care. The key global theme to emerge from the interviews related to 'managing risk' within primary care:

> *"It's quite a tricky, nebulous area* […]. *The nature of general practice is that we're dealing every day with uncertainty…"* (Interview 6, Participant 3)

A key concern of staff was that policymakers and those implementing cancer initiatives did not fully understand how risk was managed within primary care. Cancer was only one priority and there was an abundance of initiatives for a variety of conditions which primary care staff were expected to implement. Three underpinning organising themes (and associated basic themes) of 'complexity', 'continuity' and 'conflict' highlighted the tensions and difficulties that primary care face in managing the risk of early detection and referral for cancer symptoms whilst dealing with complex symptoms and care systems, patient-led factors and target-focused care. An overview of the organising and basic themes is presented in figure 1. These themes are described and discussed, contextualised by participant quotes below.

Complexity

This theme highlighted the complexity of managing risk in early cancer diagnosis because of external factors including cancer symptom differentiation and the restrictions imposed by referral criteria; the multitude of services and professionals involved in diagnostic and

For peer review only - http://bmjopen.bmj.com/site/about/guidelines.xhtml

assessment services and the plethora of policies and initiatives targeted at primary care practice.

Cancer disease and symptoms: Cancer diagnosis was an important priority area in primary care but diagnosis presented complex challenges. Cancer was a rare diagnosis in primary care although symptoms associated with cancer were common.

> *"We get lots of sore throats, and yet we get one tonsillar cancer every three* [years] *so sorting out the wheat from the chaff is a real challenge."* (Interview 3, Participant 3)

These complexities of diagnosis were compounded by what were considered rigid referral criteria, based on disease prevalence amongst those with symptoms, which led to cases being '*bounced back'* if they failed to meet diagnostic criteria. In attempts to manage what they considered was a risk to patients, some participants said, on occasion, they had to '*fudge',* '*embellish'* or '*bend-the-rules'* to ensure assessment of patients for whom they had concerns:

> *"Even though it says on the form, "don't fill out this form unless they tick any boxes" but you find a box to tick, and usually for very good reason. And I think you'd only go, slightly over egging the presentation if you were pretty sure there was something there, something going on."* (Interview 4, Participant 4)

Fragmentation and access to diagnostic services: Primary care staff expressed difficulties accessing diagnostic services due to on-going service re-configurations and the involvement of multiple agencies. This led to fragmentation in terms of staff not always knowing who, or to which services, referrals could be made.

For peer review only - http://bmjopen.bmj.com/site/about/guidelines.xhtml

There were also some concerns about fragmented relationships between primary and secondary care with several participants feeling frustrated by restricted access to diagnostics for certain conditions. Some participants considered this was due to *'empire building'* by professionals justifying and *'preserving'* their service by retaining ownership of who was qualified to make referrals:

> *"I can send people for a CT scan if I have a concern about them, for some conditions, but not others. Well why not? I'm the one who's initiating the referral in the first place. You trust me to initiate the referral to pick the patient and prepare them so that you can come along and just arrange the scan and look at it."* (Interview 4, Participant 3)

Whilst some GPs wanted more direct access to diagnostics, this was not universal, and others highlighted the need for *'training'* prior to referrals being made.

Guidelines content and information overload: Participants referred to the usefulness of guidelines to help symptom differentiation and manage risk. Knowledge was felt to be experientially derived and referral guidelines only considered useful for less experienced doctors. Risk assessment tools were occasionally considered unnecessarily complex when patients had 'red flag' symptoms and, as previously mentioned, sometimes restrictive for use in primary care:

> *"I mean, you see, from a GPs point of view, if somebody comes in who is fifty-five years of age, and passes blood in his stool, you need to exclude a cancer. Now, I don't*

For peer review only

want to know how many percentage of those people pass blood in the motion will have cancer, or how many will have piles, or inflammatory bowel disease or what have you." (Interview 4, Participant 3)

Concerns were expressed about the number of guidelines available for different conditions and inconsistencies between different sources:

"And that's the problem 'cause last year there was a big campaign, "if you have a cough for more than three weeks you need to go and see a doctor", but NICE guidelines is six weeks." (Interview 6, Participant 4)

Participants described how the *'tsunami'* of new guidelines, care plans and initiatives meant it was difficult to keep up-to-date:

"And I thought 'that's probably a squamous cell carcinoma. That needs a two-week wait referral'. Then, I actually checked the guidelines, the guidelines say that "it's got to be over one centimetre and you've got to wait more than eight weeks really for it to grow" etcetera, etcetera […] *and those things will change on a regular basis. Now, how do you expect an entire network of primary care physicians to stay current with all those guidelines and apply them religiously?"* (Interview 2, Participant 3).

Continuity

Practice staff expressed concern that a lack of continuity increased the risk of missing diagnoses and/or supporting the patient through a difficult period. This was highlighted

For peer review only - http://bmjopen.bmj.com/site/about/guidelines.xhtml

through discussions about relationships between patients and clinicians; delays in information sharing across the primary and secondary care interface and patient follow-up after initial consultation.

Patient-practice relationships: Continuity-of-carer could not always be achieved in practice, even though it was felt that patients preferred it and its absence was thought to possibly contribute to diagnostic delay:

> *"People have been hopping around from one clinician to another and that, and you don't see the evolution of the story until it hits you in the face. Patients book on the day, they don't necessarily get in the person they normally see, they get in with the available, so that can disjoint things."* (Interview 3, Participant 4)

Staff felt that smaller, rural practices had closer relationships with their patients and their community, which would lead to earlier consultation. However, others felt awareness of cancer deaths was heightened in close-knit communities, which could reinforce negative views towards cancer and cancer survivorship amongst the local population and delay access to services.

Primary care and secondary care interface: Expedited assessment following GP referral of patients through the two-week wait initiative was perceived to work well by practice staff, but delays further on in the assessment process, and outside of primary care control, were felt to put patients at risk again and exacerbated their concerns:

For peer review only - http://bmjopen.bmj.com/site/about/guidelines.xhtml

"And, well, it's very difficult from our point of view, 'cause we're saying, "look, you know this may be nothing serious, however, I want to refer you on the system, you'll get an appointment in two weeks", and they do get seen, but then there's a massive delay, so, you know, then they're kinda all heightened up because they're thinking, "{whispered} the doctor thinks I've got cancer" and then nothing happens for ages, and it's really hard for the patients." (Interview 3, Participant 5)

Practice-based follow-up: Patients themselves were also considered to cause delays if they failed to attend. Practices debated the extent to which they should chase these patients up and the risks if they did not:

"In the back of their mind they know they should, but if they face the fact that they're going back, are they are going to be faced with something else? You also have to be responsible for the fact that if that doesn't stop, you must persist, and accept the fact that, if you don't, then you could, somewhere down the line end up with something so serious that it's only going to be palliative." (Interview 2, Participant 1)

Conflict

Within this theme, managing risk was related to primary care focus on targets; conflicting opinions about the role of non-clinical staff and about the worries and tensions generated by cancer awareness campaigns.

Prevention versus target driven care: It is recognised that primary care can make a significant contribution to public health through promotion, in the practice population, of healthier

For peer review only - http://bmjopen.bmj.com/site/about/guidelines.xhtml

lifestyles and earlier presentation of symptoms of diseases, where early intervention reduces morbidity and mortality, such as cancer. Primary care staff considered cancer a public health priority but some felt that there was a tension between adopting preventative approaches in the practice such as promoting cancer awareness and early presentation and the way that primary care is currently funded through achieving specific targets mainly relating to the management of long-term conditions (Quality and Outcomes Framework):

> *"We're not as good with public health, with local public health initiatives, as maybe we should be necessarily. It's just time to do things, and those things aren't required of our core business, and when you have a very detailed contract that tells you what you will be paid for doing…"* (Interview 2, Participant 3)

Staff roles: There were conflicting views around the role of other practice staff in managing the risk of cancer detection and awareness, in particular with regard to reception staff. Some staff viewed the reception role as a health advisor, whereas others perceived their role to be purely administrative. Additionally, there were reservations about the ability of reception staff to relay cancer messages and how the public might feel about receiving health information from them:

> *"You're passing clinical responsibility to people who are non-clinical. It's alright for patients to speak to them, and that's fine, but, I'm not gonna use the receptionists as a source of information* […]. *I think anything clinical should be passed to the doctor full stop. I don't think there is any other role for the receptionist."* (Interview 4, Participant 3)

For peer review only - http://bmjopen.bmj.com/site/about/guidelines.xhtml

For peer review only

There were also concerns about potential litigation issues if reception staff were to give advice and how this could impact on the practice:

> *"I think it's also worth mentioning at this point, because of our roles, how they are, we get a lot of patients not happy about us supplying information, because we're not allowed to diagnose, obviously for obvious reasons, but if you was to imply something, that could come back on us twice as hard because you'd implied something that could be wrong, and therefore it is now your fault."* (Interview 1, Participant 1)

A further issue associated with staff roles in helping to manage risk was the identified benefits of having a chain of communication between all practice members. For example, receptionists being able to *'raise issues'* with the doctor if they know someone is coming in who has symptoms the doctor should be aware of but the patient *'may not say anything'*. This can also work the other way:

> *"Patients that I worry about, I will leave messages. I'll mention to reception or mention to people that I want to follow them up."* (Interview 6, Participant 3)

Others felt that the focus on the long-term conditions might provide opportunities for practice nurses to be more involved as they were often seeing patients who might be at higher risk of cancer because of their age, disease condition or lifestyle behaviours:

> *"Chronic disease, yeah, so diabetic, high blood pressure, chronic kidney disease,*

For peer review only - http://bmjopen.bmj.com/site/about/guidelines.xhtml

asthma, most patients will come and see the practice nurses for routine bloods, blood pressure, weight, everything like that. So often, GPs don't always see them, I mean they do the medication reviews, but, we flag to them anything that we're worried about." (Interview 2, Participant 4)

Some could also see a role for members of the wider practice team:

"We'd say 'well who are the district nurses already going out to?' You know, you can work with your local pharmacists." (Interview 2, Participant 2)

Cancer awareness: Although cancer fear was commonly acknowledged, staff were divided as to whether patients were afraid to consult the GP with cancer symptoms. Some felt that unhealthy lifestyle choices contributed to patient reluctance to present with cancer symptoms:

"The cancers where they feel that they may have contributed to it, like smoking, they tend to ignore because they don't want to be told that it may be their fault in a certain way, and they don't want to give up the lifestyle." (Interview 6, Participant 4)

Cancer awareness campaigns were felt to be important, even though those who presented to the practice following the campaign were more likely to be those at least risk:

"It's the people who never come to the doctors that you want to hit, not the people that were already coming in anyway, and they tend to be ones that see those adverts. But then if you can get one person who wouldn't normally come in and you catch them, then it's better." (Interview 5, Participant 1)

For peer review only - http://bmjopen.bmj.com/site/about/guidelines.xhtml

Campaigns were also felt to create extra work for all practice members. There were complaints that cancer awareness campaigns tend to run without consideration of other local or national campaigns which may be running simultaneously which increased the risk that practice capacity to safely respond to patients was compromised:

> *"It's like it's alright saying putting in place to get them in, but it's the sheer* volume*, isn't it? You can only cope with so much can't you?"* (Interview 5, Participant 1)

DISCUSSION

This study provides insights into the experiences of primary care staff who manage patients with symptoms suggestive of cancer. The overarching theme to emerge was the need to manage risk so that cancer patients had a timely diagnosis and were assessed appropriately. The associated subthemes of complexity, continuity and conflict highlighted the tensions and difficulties faced by staff when attempting to manage these risks in modern practice.

The study was small, descriptive and exploratory. However, it covered a diverse geographical area and included practices with varying levels of engagement with awareness and early diagnosis initiatives. We stratified on engagement to ensure we had a balance of perspectives so as to better inform policymakers of the possible barriers and drivers to the uptake of initiatives. The stratification was based on known engagement with the range of national and regional initiatives to promote earlier presentation, detection and referral of patients with symptoms suggestive of cancer available to the practices at that time.

For peer review only - http://bmjopen.bmj.com/site/about/guidelines.xhtml

The findings of our study suggest that national initiatives did not appear to wholly resolve issues in managing risk for all practitioners. Rather, in some cases, these initiatives were felt to introduce inherent risks which staff had to find ways to overcome. This included cancer awareness campaigns which, while felt to be very important to encourage patients who would otherwise delay diagnosis, also appeared to increase consultation rates from those at lesser risk. This placed additional burden on the practice.

There were also concerns about initiatives introduced to directly support practices. Participants highlighted timely diagnosis as an essential part of their role in cancer care. A finding reported in an Australian study.[16] This study also identified that the resources spent gaining timely access to specialist opinion were a major issue. In our study this appeared to be less of a problem because of the two-week waiting-time initiative. This initiative was universally embraced by participants as it was felt to work well in facilitating timely assessment. However, there were still concerns that lack of direct access to diagnostic investigations and poor communication between primary and secondary care put patients at risk due to extended delays, as reported in a study undertaken in Ireland.[3] Some participants in our study felt that the referral criteria for the two-week waiting-time initiative were too restrictive and this led to practitioners, on occasion, subverting the referral system to ensure that patients they considered were at risk, but who did not fit the referral criteria, could be assessed in a timely manner. Such concerns are not unfounded; one study has shown that 8% of cancer patients do not have symptoms which fit referral criteria.[18] This may be because the presenting signs and symptoms had a lower predictive value for cancer than those included in the guidelines. Nevertheless, there is high compliance with guidelines and some limited evidence that referral guidelines contribute, in part, to diagnostic delay reduction.[6, 18]

For peer review only - http://bmjopen.bmj.com/site/about/guidelines.xhtml

Diagnostic errors leading to primary care malpractice claims are common.[19] Researchers suggest that although GPs are more likely to correctly diagnose patients with cancer than miss cases, there are a disproportionate number of deaths amongst the latter.[20, 21] Diagnostic complexity is compounded by the frequency of consultations for symptoms associated with cancer, [21, 22] a fear highlighted by practitioners in this study. A study in Norway, which followed up patients presenting with cancer warning signs to their GP, suggests that patients with cancer may be missed if multiple warning signs and symptoms are not considered.[21] Retrospective studies of practice-based data have identified combinations of warning signs and symptoms which may be associated with increasing the likelihood of cancer.[23-24] These have been used to inform risk assessment tools to aid decision-making, with algorithms and probabilities of risk based on demographic characteristics, lifestyle factors, symptoms and/or attendance frequency.[11] Some have been disseminated nationally, and there is evidence from both quantitative and qualitative studies that they affect GPs' decisions to refer.[11] However, in our study, some participants had similar concerns about these tools as they did guidelines. A recent analysis of significant event audits in lung cancer highlighted how cancer can mimic other diagnoses and have atypical presentation.[7] Guidelines and risk assessment tools are analytical tools, and although statistical probabilities are based on uncertainty, they may be perceived to introduce a level of certainty which fails to encapsulate practitioners' tacit concerns about patients. Previous work in primary care decision-making has emphasised the analytical over the experiential, but recent research , including cancer diagnosis, suggests that experiential knowledge may have a role and may be more responsive to individual patients.[15, 25-27] Others have found that better diagnostic decisions appear to be made by older doctors [22] and, as commented on by some of our participants, that guidelines and risk assessment tools

For peer review only - http://bmjopen.bmj.com/site/about/guidelines.xhtml

may be more useful for newer practitioners who have yet to develop problem solving strategies.[28]

It has also been observed that, unlike in acute care, decision-making in primary care partly involves an understanding of the patient context and perspective.[29] Our study participants highlighted concerns that lack of continuity of carer may lead to diagnostic delays. Relational continuity was felt to be a particular problem in larger practices and urban settings. Others suggest that fragmentation of primary care and shorter consultation times has led to a lack of continuity-of-care or carer, which may hinder early diagnosis as cancer presentation can be complex in those with multiple co-morbidities.[16, 30, 31] In a qualitative study of lung cancer patients in New Zealand, patients felt that not always seeing the same GP could lead to delays in diagnosis due to poor follow up.[32] In a study in Denmark, perceived lack of accessibility and the doctor-patient relationship were associated with patient delay in seeking advice about cancer symptoms.[33] Others have found that confidence and trust in a doctor were more important predictors of cancer detection than ease of access and choice of preferred doctor.[34]

In our study, participants highlighted how other practice team members, such as nurses who are in more regular contact with patients with long-term conditions, could raise symptom awareness or be more alert to changes in, or new, symptoms suggestive of cancer. In the US and Canada, patient navigators are being used to support patients through the complex systems found in cancer management and care. It has also been suggested that these roles could be extended to support patients during the diagnostic, referral and assessment processes and ensure appropriate follow up of investigative results.[35] In our study, some staff felt that receptionists could act as navigators in terms of directing patients with symptoms to see the

For peer review only - http://bmjopen.bmj.com/site/about/guidelines.xhtml

GP.[36] Low levels of knowledge about some cancer symptoms has been demonstrated in non-medical staff.[37] However, our study highlighted that professional boundaries and concerns about litigation which might impede such initiatives; disclosures or discussions of diagnostic issues within a public reception location also raised ethical concerns. Research within primary care suggests that facilitation of communities of practice and interdisciplinary knowledge sharing may help to identify the role of other practice team members in promoting earlier cancer presentation and diagnosis;[38] although training needs should be addressed.[37]

This study has highlighted that a 'one-size-fits-all' approach to implementing initiatives is unlikely to succeed as practitioners expressed a range of views and opinions on the benefits of different initiatives. Lack of implementation of initiatives in primary care is not always because of resistance to the initiatives themselves, but sometimes because of the sheer number across a range of priority areas that practices are expected to implement, often simultaneously. Policymakers should consider more carefully how these impact on primary care, how they can be embedded into practice systems and emphasise and exploit synergies with other disease conditions.

For there to be greater success of initiatives aimed at promoting earlier presentation, detection and referral in primary care, there needs to be further work on understanding how primary care manage risk in the face of inherent uncertainty, organisational changes and competing priorities.

For peer review only - http://bmjopen.bmj.com/site/about/guidelines.xhtml

ACKNOWLEDGEMENTS: Our thanks to the practices who participated in this study and to the staff of the Lancashire and South Cumbria Cancer Network who assisted us.

COMPETING INTERESTS: PD was public health lead for LSCCN and NC is part-funded by a grant from LSCCN; PD was also funded by LSCCN to undertake evaluative work.

FUNDING: This work was supported by Lancashire and South Cumbria Cancer Network (LSCCN) as part of the National Cancer Awareness and Early Detection initiative (NAEDI) funding 'cancer networks supporting primary care'.

CONTRIBUTIONS:

NC recruited participants, carried out interviews, transcribed the interviews, coded the transcripts, contributed to the development of the coding framework, and contributed to the writing of the manuscript.

GT carried out interviews, coded the transcripts, contributed to the development of the coding framework and contributed to the writing of the manuscript.

PD designed the study, collated practice engagement data, contributed to the development of the coding of the framework and contributed to the writing of the manuscript.

DATA SHARING: No further data are available

REFERENCES

For peer review only

1. Haward R. The Calman-Hine report: a personal retrospective on the UK's first comprehensive policy on cancer services. *The Lancet Oncology*. 2006;7(4):336-46.
2. Coleman MP, Foreman D, Bryant H, et al. Cancer survival in Australia, Canada, Denmark, Norway, Sweden, and the UK, 1995—2007 (the International Cancer Benchmarking Partnership): an analysis of population-based cancer registry data. *The Lancet*. 2011;377:127–38. doi:10.1016/S0140-6736(10)62231-3.
3. Daly H, Collins C. Barriers to early diagnosis of cancer in primary care: a needs assessment of GPs. *Ir Med J*. 2007; 100(10):624-26
4. Ingebrigsten SG, Scheel BI, Hart B, et al. Frequency of 'warning signs of cancer' in Norwegian general practice, with prospective recording of subsequent cancer. *Fam Pract*. 2013;30:153-160
5. National Institute for Clinical Excellence. Clinical Guideline 27. Referral guidelines for suspected cancer. 2005. http://www.nice.org.uk/nicemedia/live/10968/29814/29814.pdf accessed 3/1/2014.
6. Neal RD, Din NU, Hamilton W, et al. Comparison of cancer diagnostic intervals before and after implementation of NICE guidelines: analysis of data from the UK General Practice Research Database. *Br J Cancer*. 2014;110(3):584-92. doi:10.1038/bjc.2013.791. 4.
7. Mitchell ED, Rubin G, McLeod U. Understanding diagnosis of lung cancer in primary care: qualitative synthesis of significant event audit reports. *Br J Gen Pract*. 2013;63(606):e37-46. doi:10.3399/bjgp13X660760.
8. Royal College of General Practitioners. Clinical Innovation and Research Centre. National Audit of Cancer Diagnosis in Primary Care. 2011. http://www.rcgp.org.uk/news/2011/november/~/media/Files/News/National_Audit_of_Cancer_Diagnosis_in_Primary-Care.ashx. last accessed 03/01/2014.

For peer review only - http://bmjopen.bmj.com/site/about/guidelines.xhtml

9. Meechan D, Gildea C, Hollingworth L, et al. Variation in use of the 2-week referral pathway for suspected cancer: A cross-sectional analysis. *Br J Gen Pract*. 2012; 62(602):e590–97. doi:10.3399/bjgp12X654551.

10. Elliss-Brookes L, McPhail S, Ives A, et al. Routes to diagnosis for cancer - determining the patient journey using multiple routine data sets. *Br J Cancer*. 2012;107(8):1220-26. doi:10.1038/bjc.2012.408.

11. Hamilton W, Green T, Martins T et al. Evaluation of risk assessment tools for suspected cancer in general practice: a cohort study. *Br J Gen Pract*. 2013;63(606):e30-36. doi:10.3399/bjgp13X660751.

12. Emery JD, Walter FM, Gray V, et al. Diagnosing cancer in the bush: a mixed-methods study of symptom appraisal and help-seeking behaviour in people with cancer from rural Western Australia. *Fam Pract*. 2013 Jun;30(3):294-301.doi: 10.1093/fampra/cms087

13. Pedersen AF, Hansen RP, Vedsted P. Patient delay in colorectal cancer patients: associations with rectal bleeding and thoughts about cancer. *PLoS One*. 2013:Jul 22;8(7):e69700.doi: 10.1371/journal.pone.0069700

14. Marlow LA, McGregor LM, Nazroo JY, et al. Facilitators and barriers to help-seeking for breast and cervical cancer symptoms: a qualitative study with an ethnically diverse sample in London. *Psychooncology*. 2013;Dec 19.doi:10.1002/pon.3464

15. Johansen ML, Holtedahl KA, Rudebeck CE. How does the thought of cancer arise in a general practice consultation? Interviews with GPs. *Scand J Prim Health Care*. 2012;30(3):135-40. doi:10.3109/02813432.2012.688701.

16. Mitchell GK, Burridge LH, Colquist SP, et al. General Practitioners' perceptions of their role in cancer care and factors which influence this role. *Health Soc Care Community*. 2012; 20(6):607-16. doi:10.1111/j.1365-2524.2012.01075.x

For peer review only - http://bmjopen.bmj.com/site/about/guidelines.xhtml

17. Attride-Stirling J. Thematic networks: an analytic tool for qualitative research. *Qualitative Research*. 2001;1(3):385-405. doi:10.1177/146879410100100307.

18. Baughan P, Keatings J, O'Neill B. Urgent suspected cancer referrals from general practice: audit of compliance with guidelines and referral outcomes. *Br J Gen Pract*. 2011; doi:10.3399/bjgp11X606591.

19. Wallace E, Lowry J, Smith SM, et al. The epidemiology of malpractice claims in primary care: a systematic review. *BMJ Open*. 2013;3:e002929. doi:10.1136/bmjopen-2013-002929.

20. Balla J, Heneghan C, Thompson M, et al. Clinical decision making in a high-risk primary care environment: a qualitative study in the UK. *BMJ Open*. 2012;2:e000414. doi:10.1136/bmjopen-2011-000414

21. Scheel BI, Ingebrigsten SG, Thorsen T, et al. Cancer suspicion in general practice: the role of symptoms and patient characteristics, and their association with subsequent cancer. *Br J Gen Pract*. 2013;doi:10.3399/bjgp13X671614

22. Jiwa M, Gordon M, Arnet H, et al. Referring patients to specialists: a structured vignette survey of Australian and British GPs. *BMC Fam Pract*. 2008; 9:2.doi: 10.1186/1471-2296-9-2.

23. Emery J. Editorial: Assessment of cancer risk in men and women. *Br J Gen Pract*. 2013;63(606):4-5.doi:10.3399/bjgp13X660607.

24. Hamilton W. The CAPER studies: five case-control studies aimed at identifying and quantifying the risk of cancer in symptomatic primary care patients. *Br J Cancer*.2009;101: S80-S86 doi:10.1038/sj.bjc.6605396

25. André M, Andén A, Borgquist L, et al. GPs' decision-making-perceiving the patient as a person or a disease. *BMC Fam Pract*. 2012;13:38. doi:10.1186/1471-2296-13-38.

For peer review only - http://bmjopen.bmj.com/site/about/guidelines.xhtml

26. Stolper E, Van de Wiel M, Van Royen P, et al. Gut feelings as a third track in general practitioners' diagnostic reasoning. *J Gen Intern Med*. 2011;26(2):197-203. doi:10.1007/s11606-010-1524-5.

27. Zwolsman S, te Pas E, Hooft L, et al. Barriers to GPs' use of evidence-based medicine: a systematic review. *Br J Gen Pract*. 2012;62(600):e511-21. doi: 10.3399/bjgp12X652382.

28. Salah A, Hassan A. Cognitive schemes and strategies in diagnostic and therapeutic decision making: a primer for trainees. *Perspect Med Educ.* 2013; 2:321-31. doi:10.1007/s40037-013-0070-3

29. O'Riordan M, Dahinden A, Aktürk Z, et al. Dealing with uncertainty in general practice: an essential skill for the general practitioner. *Qual Prim Care*. 2011;19:175-81.

30. Brindle L, Pope C, Corner J, et al. Eliciting symptoms interpreted as normal by patients with early lung cancer. *BMJ Open*. 2012;2:e001977. doi:10.1136/bmjopen-2012-001977.

31. Round T, Steed L, Shankleman J, et al. Primary care delays in diagnosing cancer: what is causing them and what can we do about them? *J R Soc Med*. 2013;106(11):437-40. doi:10.1177/0141076813504744.

32. Walton L, McNeill R, Stevens W, et al. Patient perceptions of barriers to the early diagnosis of lung cancer and advice for health service improvement. *Fam Pract*. 2013;30:439-44 doi:10.1093/fampra/cmt001

33. Hansen RP, Vedsted P, Sokolowsk I, et al. General practitioner characteristics and delay in cancer diagnosis: a population-based cohort study. *BMC Fam Pract*. 2011; 12:100

For peer review only - http://bmjopen.bmj.com/site/about/guidelines.xhtml

34. Rogers S, Gildea C, Meechan D, et al. Access, continuity of care and consultation quality: which best predicts urgent cancer referrals from general practice? *J Public Health (Oxf).* 2014 Jan 23. [Epub ahead of print]

35. Lee J-H, Fulp W, Wells KJ, et al. Effect of patient navigation on time to diagnostic resolution among patients with colorectal cancer-related abnormalities. *J Canc Educ.* 2014;29:144-50

36. Dohan D, Schrag D. Using navigators to improve care of underserved patients. *Cancer*. 2005;104:848-55. doi:10.1002/cncr.21214.

37. Cook N, Hart A, Nuttall K, et al. A telephone survey of cancer awareness among frontline staff: informing training needs. *Br J Cancer*. 2011;105(3):340-45. doi:10.1038/bjc.

38. Kislov R, Walshe K, Harvey G. Managing boundaries in primary care service improvement: a developmental approach to communities of practice. *Implement Sci*. 2012;7:97. doi:10.1186/1748-5908-7-97.

For peer review only

Figure 1: organising themes of complexity, continuity and conflict with their associated basic themes, centred around the global theme of managing risk in primary care

For peer review only

For peer review only - http://bmjopen.bmj.com/site/about/guidelines.xhtml

Box 1: Semi-structured interview schedule

- Why do you think people with symptoms suggestive of cancer do not present, get seen or diagnosed or referred earlier?
- What do you know about the initiatives that concern the earlier presentation, diagnosis and referral of cancer symptoms?
  - Prompts around specific tools/initiatives/referral criteria
- Have you accessed any local training events concerning the identification, referral of cancer patients?
  - Prompts around specific training attended, and how learning is usually undertaken
- Overall, what is working well in terms of the implementation and use of these initiatives?
- Overall, have you experienced any/or what do you consider to be the main barriers in the implementation/access/use of these various initiatives?
- Are there any practice based issues that may affect the early identification and referral for cancer patients?
  - Prompts around staff and practice issues, communication, administration
- Overall, what do you think local practices could do to help promote and diagnose cancer symptoms?
  - Prompts around team roles and other practice issues
- Do you know about the Lancashire and South Cumbria Cancer Network and what their role is?
  - Prompts around extent of engagement, attitudes and areas for improvement
- What is your opinion concerning the forthcoming ‘access to diagnostics’ initiative (initiative for practices to make direct referrals to diagnostics such as x-rays, CT scans, ultrasound)?
  - Prompts around who should make the referral
- Are there any additional support mechanisms/external to the practice that need to be in place to help with promotion/diagnosis and referral?
- Any further issues or concerns you would like to raise about this work?

For peer review only - http://bmjopen.bmj.com/site/about/guidelines.xhtml

Managing the risk of cancer earlier presentation, detection and referral: a qualitative study of primary care staff views

For peer review only

Neil Cook, Research Assistant, School of Medicine and Dentistry, University of Central Lancashire, Preston, England*

Gillian Thomson, Senior Research Fellow, School of Health, University of Central Lancashire, Preston, England

Paola Dey, Professor of Public Health Epidemiology, School of Medicine and Dentistry, University of Central Lancashire, Preston, England

*corresponding author: Neil Cook, School of Medicine and Dentistry, Greenbank Building, University of Central Lancashire, Adelphi Street, Preston, England PR1 2HE

Email: NCook2@uclan.ac.uk Phone: 01772 893409

Key Words: primary health care, general practice, neoplasms, qualitative research, early detection of cancer.

Word count: abstract- 232, manuscript - 4,504

For peer review only - http://bmjopen.bmj.com/site/about/guidelines.xhtml

ABSTRACT

Objectives: In the UK, there have been a number of national initiatives to promote earlier detection and prompt referral of patients presenting to primary care with signs and symptoms of cancer. The aim of the study was to explore the experiences of a range of primary care staff in promoting earlier presentation, detection and referral of patients with symptoms suggestive of cancer.

Setting: Six primary care practices in North West England.

Participants: Thirty-nine primary care staff from a variety of disciplines took part in five group and four individual interviews.

Results: The global theme to emerge from the interviews was 'managing risk', which had three underpinning organising themes: 'complexity', relating to uncertainty of cancer diagnoses, service fragmentation and plethora of guidelines; 'continuity', relating to relationships between practice staff and their patients and between primary and secondary care; 'conflict' relating to policy drivers and staff role boundaries. A key concern of staff was that policymakers and those implementing cancer initiatives did not fully understand how risk was managed within primary care.

Conclusion: Primary care staff expressed a range of views and opinions on the benefits of cancer initiatives. National initiatives did not appear to wholly resolve issues in managing risk for all practitioners. Staff were concerned about the number of guidelines and priorities

For peer review only

For peer review only - http://bmjopen.bmj.com/site/about/guidelines.xhtml

they were expected to implement. These issues need to be considered by policymakers when developing and implementing new initiatives.

ARTICLE SUMMARY

Strengths and limitations of this study

- The study investigates the experiences of a range of primary care staff around implementing initiatives for the earlier diagnosis of cancer.
- The underlying concern in primary care is related to managing risk.
- The sample included a mix of practices with different practice characteristics and included those known to be engaged in national and regional initiatives and those who were less engaged.
- The sample was drawn from a single English region.

For peer review only

For peer review only - http://bmjopen.bmj.com/site/about/guidelines.xhtml

INTRODUCTION

Studies in the 1990s showed that UK cancer survival rates were worse than many other European countries, following which there has been two decades of concerted effort to expedite access to proven effective cancer treatments.[1] While there have been improvements in cancer survival latterly, the UK still lags behind many other countries with similar health care systems, which may be partly due to later stage of disease at presentation.[2]

Both nationally and internationally, primary care is seen to have a key role in improving cancer survival by reducing delays in diagnosis through promoting earlier presentation and through earlier detection and referral of those with symptoms for further specialist assessment.[3, 4] In the UK, national campaigns extol those with symptoms to see their general practitioner earlier; there are national referral guidelines for suspected cancer and a national system for urgent referral from primary to secondary care (two-week waiting-time initiative).[5] However, evidence suggests that there may be further room for improvement:[6] there is practice variation in the use of the 'two-week' initiative and some patients are seen several times in primary care before referral.[7-9] A significant proportion of patients also present through emergency routes and have poorer survival.[10]

To further support primary care in the UK, resources were developed and/or disseminated by the Department of Health's National Awareness and Early Diagnosis Initiative (NAEDI), established in 2008, and the Royal College of General Practitioners (RCGP) including: audit and significant events analysis tools,[7, 8] general practitioner (GP) level cancer profile data, safety netting recommendations and risk assessment tools.[11] Key initiatives have mainly

For peer review only - http://bmjopen.bmj.com/site/about/guidelines.xhtml

For peer review only

focused on GPs, but other members of the primary care team may have key roles: an area which has been largely unexplored.

A number of international studies have been undertaken in attempts to understand the reasons behind delay in cancer diagnosis. Qualitative studies have mostly focussed on patient perspectives.[12-14] Only a few have explored primary care experiences and these have been mainly limited to decision-making processes or referral pathways [11, 15, 16] and from the perspective of the general practitioner. [3, 11, 15,16] In order to understand how a range of initiatives across the patient pathway in primary care could be more effective and the role of other members of the practice team, we explored the experiences of a range of primary care staff in supporting earlier presentation, detection and referral of those with symptoms suggestive of cancer. The study was undertaken in one region of England, which at that time was covered by the Lancashire and South Cumbria Cancer Network (LSCCN).

METHODS

This was a qualitative descriptive study utilising both individual and group-based interviews. It aimed to recruit staff from six practices who were differentially engaged with the national awareness and early diagnosis of cancer agenda. GP practices within LSSCN were stratified into one of three groups at the end of June 2012. High engagers had participated in at least one of the following: RCGP cancer audit, attendance at a course on early diagnosis or face-to-face meetings to discuss GP cancer profiles and action planning. Medium engagers had attended at least one regional meeting about cancer awareness. Low engagers were not

For peer review only - http://bmjopen.bmj.com/site/about/guidelines.xhtml

known to have engaged in any initiatives. Of 254 practices in the geographical area, there were 51 high engagers, 69 medium engagers and 134 low engagers.

Within each group, a random sample of 10 practices were sent a letter about the study, followed up with a phone call from the research team one week later. The initial aim was to recruit at least two practices in each stratum. Due to low uptake within the medium and low engager strata, these categories were merged and a random sample of a further 15 practices sent letters. Practices were offered a choice of either group or individual interviews as it was recognised that time constraints prevent some practice staff from taking part in group interviews and some may feel uncomfortable discussing the issues with colleagues. Individual interviews were the preferred method in only one practice. Interviews (group and individual) were held on only one occasion in each practice.

Six practices agreed to take part in semi-structured, audio-recorded interviews, which were attended by two researchers, between September and October 2012. We recruited three practices in the high stratum and three practices in the merged medium and low stratum (a mix of low and medium engagers). The topic guide is outlined in Box 1. Thirty-nine participants took part in group (n=5) or individual (n=4) interviews. Job roles included GP (n=9), receptionist (n=7), nurse (n=6), manager (n=6), secretary (n=5), health care assistant (n=3), medical student (n=2) and phlebotomist (n=1). Practice characteristics are shown in table 1.

For peer review only - http://bmjopen.bmj.com/site/about/guidelines.xhtml

Table 1: Practice and interview characteristics

Practice characteristic	No. of practices
No of partners	
4 or more	3
3 or less	3
Deprivation Quintile*	
5 or 4 (more deprived)	3
2 or 3	3
1 (most affluent)	0
Population Density**	
<15%	2
15 to 39%	2
>=40%	2

*based on index of multiple deprivation of practice location 2010 (source: Department for Communities and Local Government, Indices of Deprivation 2010)
** Person per hectare based on practice location (source: ONS Neighbourhood Statistics)

The interviews took between 38 and 67 minutes to complete, and between four and eleven staff took part in each practice. All transcribed data were entered into NVivo 10 and analysed using a Thematic Network Analysis approach.[17] This involved an iterative and cyclical process of reading and analysis to identify basic, organising and global themes within the dataset. Analysis was undertaken by two authors (GT and NC) independently on six transcripts initially, followed by an in-depth discussion and consensual validation of key themes. A further cycle of independent and collaborative analysis was then undertaken on a further sub-set of transcripts to ensure rigour and authenticity of the themes generated. All thematic decisions were discussed with the third author (PD). The study was approved by the University of Central Lancashire ‘STEMH’ ethics committee and individual written informed consent was obtained from all participants.

For peer review only - http://bmjopen.bmj.com/site/about/guidelines.xhtml

RESULTS

Overall, practice staff were well aware of the two-week waiting-time initiative and had good knowledge about the national 'Be Clear on Cancer' cancer awareness public campaigns, but had less awareness about other initiatives specifically targeted at primary care. The key global theme to emerge from the interviews related to 'managing risk' within primary care:

> *"It's quite a tricky, nebulous area* […]. *The nature of general practice is that we're dealing every day with uncertainty…"* (Interview 6, Participant 3)

A key concern of staff was that policymakers and those implementing cancer initiatives did not fully understand how risk was managed within primary care. Cancer was only one priority and there was an abundance of initiatives for a variety of conditions which primary care staff were expected to implement. Three underpinning organising themes (and associated basic themes) of 'complexity', 'continuity' and 'conflict' highlighted the tensions and difficulties that primary care face in managing the risk of early detection and referral for cancer symptoms whilst dealing with complex symptoms and care systems, patient-led factors and target-focused care. An overview of the organising and basic themes is presented in figure 1. These themes are described and discussed, contextualised by participant quotes below.

Complexity

This theme highlighted the complexity of managing risk in early cancer diagnosis because of external factors including cancer symptom differentiation and the restrictions imposed by

For peer review only - http://bmjopen.bmj.com/site/about/guidelines.xhtml

referral criteria; the multitude of services and professionals involved in diagnostic and assessment services and the plethora of policies and initiatives targeted at primary care practice.

Cancer disease and symptoms: Cancer diagnosis was an important priority area in primary care but diagnosis presented complex challenges. Cancer was a rare diagnosis in primary care although symptoms associated with cancer were common.

> *"We get lots of sore throats, and yet we get one tonsillar cancer every three* [years] *so sorting out the wheat from the chaff is a real challenge."* (Interview 3, Participant 3)

These complexities of diagnosis were compounded by what were considered rigid referral criteria, based on disease prevalence amongst those with symptoms, which led to cases being '*bounced back*' if they failed to meet diagnostic criteria. In attempts to manage what they considered was a risk to patients, some participants said, on occasion, they had to '*fudge*', '*embellish*' or '*bend-the-rules*' to ensure assessment of patients for whom they had concerns:

> *"Even though it says on the form, "don't fill out this form unless they tick any boxes" but you find a box to tick, and usually for very good reason. And I think you'd only go, slightly over egging the presentation if you were pretty sure there was something there, something going on."* (Interview 4, Participant 4)

Fragmentation and access to diagnostic services: Primary care staff expressed difficulties accessing diagnostic services due to on-going service re-configurations and the involvement

For peer review only - http://bmjopen.bmj.com/site/about/guidelines.xhtml

of multiple agencies. This led to fragmentation in terms of staff not always knowing who, or to which services, referrals could be made.

There were also some concerns about fragmented relationships between primary and secondary care with several participants feeling frustrated by restricted access to diagnostics for certain conditions. Some participants considered this was due to *'empire building'* by professionals justifying and *'preserving'* their service by retaining ownership of who was qualified to make referrals:

> *"I can send people for a CT scan if I have a concern about them, for some conditions, but not others. Well why not? I'm the one who's initiating the referral in the first place. You trust me to initiate the referral to pick the patient and prepare them so that you can come along and just arrange the scan and look at it."* (Interview 4, Participant 3)

Whilst some GPs wanted more direct access to diagnostics, this was not universal, and others highlighted the need for *'training'* prior to referrals being made.

Guidelines content and information overload: Participants referred to the usefulness of guidelines to help symptom differentiation and manage risk. Knowledge was felt to be experientially derived and referral guidelines only considered useful for less experienced doctors. Risk assessment tools were occasionally considered unnecessarily complex when patients had 'red flag' symptoms and, as previously mentioned, sometimes restrictive for use in primary care:

For peer review only - http://bmjopen.bmj.com/site/about/guidelines.xhtml

> *"I mean, you see, from a GPs point of view, if somebody comes in who is fifty-five years of age, and passes blood in his stool, you need to exclude a cancer. Now, I don't want to know how many percentage of those people pass blood in the motion will have cancer, or how many will have piles, or inflammatory bowel disease or what have you."* (Interview 4, Participant 3)

Concerns were expressed about the number of guidelines available for different conditions and inconsistencies between different sources:

> *"And that's the problem 'cause last year there was a big campaign, "if you have a cough for more than three weeks you need to go and see a doctor", but NICE guidelines is six weeks.*" (Interview 6, Participant 4)

Participants described how the *'tsunami'* of new guidelines, care plans and initiatives meant it was difficult to keep up-to-date:

> *"And I thought 'that's probably a squamous cell carcinoma. That needs a two-week wait referral'. Then, I actually checked the guidelines, the guidelines say that "it's got to be over one centimetre and you've got to wait more than eight weeks really for it to grow" etcetera, etcetera* […] *and those things will change on a regular basis. Now, how do you expect an entire network of primary care physicians to stay current with all those guidelines and apply them religiously?"* (Interview 2, Participant 3).

Continuity

For peer review only - http://bmjopen.bmj.com/site/about/guidelines.xhtml

Practice staff expressed concern that a lack of continuity increased the risk of missing diagnoses and/or supporting the patient through a difficult period. This was highlighted through discussions about relationships between patients and clinicians; delays in information sharing across the primary and secondary care interface and patient follow-up after initial consultation.

Patient-practice relationships: Continuity-of-carer could not always be achieved in practice, even though it was felt that patients preferred it and its absence was thought to possibly contribute to diagnostic delay:

> *"People have been hopping around from one clinician to another and that, and you don't see the evolution of the story until it hits you in the face. Patients book on the day, they don't necessarily get in the person they normally see, they get in with the available, so that can disjoint things."* (Interview 3, Participant 4)

Staff felt that smaller, rural practices had closer relationships with their patients and their community, which would lead to earlier consultation. However, others felt awareness of cancer deaths was heightened in close-knit communities, which could reinforce negative views towards cancer and cancer survivorship amongst the local population and delay access to services.

Primary care and secondary care interface: Expedited assessment following GP referral of patients through the two-week wait initiative was perceived to work well by practice staff, but delays further on in the assessment process, and outside of primary care control, were felt to put patients at risk again and exacerbated their concerns:

For peer review only - http://bmjopen.bmj.com/site/about/guidelines.xhtml

"And, well, it's very difficult from our point of view, 'cause we're saying, "look, you know this may be nothing serious, however, I want to refer you on the system, you'll get an appointment in two weeks", and they do get seen, but then there's a massive delay, so, you know, then they're kinda all heightened up because they're thinking, "{whispered} the doctor thinks I've got cancer" and then nothing happens for ages, and it's really hard for the patients." (Interview 3, Participant 5)

Practice-based follow-up: Patients themselves were also considered to cause delays if they failed to attend. Practices debated the extent to which they should chase these patients up and the risks if they did not:

"In the back of their mind they know they should, but if they face the fact that they're going back, are they are going to be faced with something else? You also have to be responsible for the fact that if that doesn't stop, you must persist, and accept the fact that, if you don't, then you could, somewhere down the line end up with something so serious that it's only going to be palliative." (Interview 2, Participant 1)

Conflict

Within this theme, managing risk was related to primary care focus on targets; conflicting opinions about the role of non-clinical staff and about the worries and tensions generated by cancer awareness campaigns.

Prevention versus target driven care: It is recognised that primary care can make a significant

For peer review only - http://bmjopen.bmj.com/site/about/guidelines.xhtml

contribution to public health through promotion, in the practice population, of healthier lifestyles and earlier presentation of symptoms of diseases, where early intervention reduces morbidity and mortality, such as cancer. Primary care staff considered cancer a public health priority but some felt that there was a tension between adopting preventative approaches in the practice such as promoting cancer awareness and early presentation and the way that primary care is currently funded through achieving specific targets mainly relating to the management of long-term conditions (Quality and Outcomes Framework):

> *"We're not as good with public health, with local public health initiatives, as maybe we should be necessarily. It's just time to do things, and those things aren't required of our core business, and when you have a very detailed contract that tells you what you will be paid for doing…"* (Interview 2, Participant 3)

Staff roles: There were conflicting views around the role of other practice staff in managing the risk of cancer detection and awareness, in particular with regard to reception staff. Some staff viewed the reception role as a health advisor, whereas others perceived their role to be purely administrative. Additionally, there were reservations about the ability of reception staff to relay cancer messages and how the public might feel about receiving health information from them:

> *"You're passing clinical responsibility to people who are non-clinical. It's alright for patients to speak to them, and that's fine, but, I'm not gonna use the receptionists as a source of information* […]. *I think anything clinical should be passed to the doctor*

For peer review only

full stop. I don't think there is any other role for the receptionist." (Interview 4, Participant 3)

There were also concerns about potential litigation issues if reception staff were to give advice and how this could impact on the practice:

"I think it's also worth mentioning at this point, because of our roles, how they are, we get a lot of patients not happy about us supplying information, because we're not allowed to diagnose, obviously for obvious reasons, but if you was to imply something, that could come back on us twice as hard because you'd implied something that could be wrong, and therefore it is now your fault." (Interview 1, Participant 1)

A further issue associated with staff roles in helping to manage risk was the identified benefits of having a chain of communication between all practice members. For example, receptionists being able to *'raise issues'* with the doctor if they know someone is coming in who has symptoms the doctor should be aware of but the patient *'may not say anything'*. This can also work the other way:

"Patients that I worry about, I will leave messages. I'll mention to reception or mention to people that I want to follow them up." (Interview 6, Participant 3)

Others felt that the focus on the long-term conditions might provide opportunities for practice nurses to be more involved as they were often seeing patients who might be at higher risk of cancer because of their age, disease condition or lifestyle behaviours:

For peer review only - http://bmjopen.bmj.com/site/about/guidelines.xhtml

"Chronic disease, yeah, so diabetic, high blood pressure, chronic kidney disease, asthma, most patients will come and see the practice nurses for routine bloods, blood pressure, weight, everything like that. So often, GPs don't always see them, I mean they do the medication reviews, but, we flag to them anything that we're worried about." (Interview 2, Participant 4)

Some could also see a role for members of the wider practice team:

"We'd say 'well who are the district nurses already going out to?' You know, you can work with your local pharmacists." (Interview 2, Participant 2)

Cancer awareness: Although cancer fear was commonly acknowledged, staff were divided as to whether patients were afraid to consult the GP with cancer symptoms. Some felt that unhealthy lifestyle choices contributed to patient reluctance to present with cancer symptoms:

"The cancers where they feel that they may have contributed to it, like smoking, they tend to ignore because they don't want to be told that it may be their fault in a certain way, and they don't want to give up the lifestyle." (Interview 6, Participant 4)

Cancer awareness campaigns were felt to be important, even though those who presented to the practice following the campaign were more likely to be those at least risk:

"It's the people who never come to the doctors that you want to hit, not the people that were already coming in anyway, and they tend to be ones that see those adverts.

For peer review only - http://bmjopen.bmj.com/site/about/guidelines.xhtml

But then if you can get one person who wouldn't normally come in and you catch them, then it's better." (Interview 5, Participant 1)

Campaigns were also felt to create extra work for all practice members. There were complaints that cancer awareness campaigns tend to run without consideration of other local or national campaigns which may be running simultaneously which increased the risk that practice capacity to safely respond to patients was compromised:

"It's like it's alright saying putting in place to get them in, but it's the sheer volume, *isn't it? You can only cope with so much can't you?"* (Interview 5, Participant 1)

DISCUSSION

This study provides insights into the experiences of primary care staff who manage patients with symptoms suggestive of cancer. The overarching theme to emerge was the need to manage risk so that cancer patients had a timely diagnosis and were assessed appropriately. The associated subthemes of complexity, continuity and conflict highlighted the tensions and difficulties faced by staff when attempting to manage these risks in modern practice.

The study was small, descriptive and exploratory. However, it covered a diverse geographical area and included practices with varying levels of engagement with awareness and early diagnosis initiatives. We stratified on engagement to ensure we had a balance of perspectives so as to better inform policymakers of the possible barriers and drivers to the uptake of initiatives. The stratification was based on known engagement with the range of national and

For peer review only - http://bmjopen.bmj.com/site/about/guidelines.xhtml

regional initiatives to promote earlier presentation, detection and referral of patients with symptoms suggestive of cancer available to the practices at that time.

The findings of our study suggest that national initiatives did not appear to wholly resolve issues in managing risk for all practitioners. Rather, in some cases, these initiatives were felt to introduce inherent risks which staff had to find ways to overcome. This included cancer awareness campaigns which, while felt to be very important to encourage patients who would otherwise delay diagnosis, also appeared to increase consultation rates from those at lesser risk. This placed additional burden on the practice.

There were also concerns about initiatives introduced to directly support practices. Participants highlighted timely diagnosis as an essential part of their role in cancer care. A finding reported in an Australian study.[16] This study also identified that the resources spent gaining timely access to specialist opinion were a major issue. In our study this appeared to be less of a problem because of the two-week waiting-time initiative. This initiative was universally embraced by participants as it was felt to work well in facilitating timely assessment. However, there were still concerns that lack of direct access to diagnostic investigations and poor communication between primary and secondary care put patients at risk due to extended delays, as reported in a study undertaken in Ireland.[3] Some participants in our study felt that the referral criteria for the two-week waiting-time initiative were too restrictive and this led to practitioners, on occasion, subverting the referral system to ensure that patients they considered were at risk, but who did not fit the referral criteria, could be assessed in a timely manner. Such concerns are not unfounded; one study has shown that 8% of cancer patients do not have symptoms which fit referral criteria.[18] This may be because the presenting signs and symptoms had a lower predictive value for cancer than those included in the guidelines. Nevertheless, there is high compliance with guidelines and some

For peer review only - http://bmjopen.bmj.com/site/about/guidelines.xhtml

limited evidence that referral guidelines contribute, in part, to diagnostic delay reduction.[6, 18]

Diagnostic errors leading to primary care malpractice claims are common.[19] Researchers suggest that although GPs are more likely to correctly diagnose patients with cancer than miss cases, there are a disproportionate number of deaths amongst the latter.[20, 21] Diagnostic complexity is compounded by the frequency of consultations for symptoms associated with cancer, [21, 22] a fear highlighted by practitioners in this study. A study in Norway, which followed up patients presenting with cancer warning signs to their GP, suggests that patients with cancer may be missed if multiple warning signs and symptoms are not considered.[21] Retrospective studies of practice-based data have identified combinations of warning signs and symptoms which may be associated with increasing the likelihood of cancer.[23-24] These have been used to inform risk assessment tools to aid decision-making, with algorithms and probabilities of risk based on demographic characteristics, lifestyle factors, symptoms and/or attendance frequency.[11] Some have been disseminated nationally, and there is evidence from both quantitative and qualitative studies that they affect GPs' decisions to refer.[11] However, in our study, some participants had similar concerns about these tools as they did guidelines. A recent analysis of significant event audits in lung cancer highlighted how cancer can mimic other diagnoses and have atypical presentation.[7] Guidelines and risk assessment tools are analytical tools, and although statistical probabilities are based on uncertainty, they may be perceived to introduce a level of certainty which fails to encapsulate practitioners' tacit concerns about patients. Previous work in primary care decision-making has emphasised the analytical over the experiential, but recent research , including cancer diagnosis, suggests that experiential knowledge may have a role and may be more responsive to individual patients.[15, 25-27]

For peer review only - http://bmjopen.bmj.com/site/about/guidelines.xhtml

Others have found that better diagnostic decisions appear to be made by older doctors [22] and, as commented on by some of our participants, that guidelines and risk assessment tools may be more useful for newer practitioners who have yet to develop problem solving strategies.[28]

It has also been observed that, unlike in acute care, decision-making in primary care partly involves an understanding of the patient context and perspective.[29] Our study participants highlighted concerns that lack of continuity of carer may lead to diagnostic delays. Relational continuity was felt to be a particular problem in larger practices and urban settings. Others suggest that fragmentation of primary care and shorter consultation times has led to a lack of continuity-of-care or carer, which may hinder early diagnosis as cancer presentation can be complex in those with multiple co-morbidities.[16, 30, 31] In a qualitative study of lung cancer patients in New Zealand, patients felt that not always seeing the same GP could lead to delays in diagnosis due to poor follow up.[32] In a study in Denmark, perceived lack of accessibility and the doctor-patient relationship were associated with patient delay in seeking advice about cancer symptoms.[33] Others have found that confidence and trust in a doctor were more important predictors of cancer detection than ease of access and choice of preferred doctor.[34]

In our study, participants highlighted how other practice team members, such as nurses who are in more regular contact with patients with long-term conditions, could raise symptom awareness or be more alert to changes in, or new, symptoms suggestive of cancer. In the US and Canada, patient navigators are being used to support patients through the complex systems found in cancer management and care. It has also been suggested that these roles could be extended to support patients during the diagnostic, referral and assessment processes

For peer review only - http://bmjopen.bmj.com/site/about/guidelines.xhtml

and ensure appropriate follow up of investigative results.[35] In our study, some staff felt that receptionists could act as navigators in terms of directing patients with symptoms to see the GP.[36] Low levels of knowledge about some cancer symptoms has been demonstrated in non-medical staff.[37] However, our study highlighted that professional boundaries and concerns about litigation which might impede such initiatives; disclosures or discussions of diagnostic issues within a public reception location also raised ethical concerns. Research within primary care suggests that facilitation of communities of practice and interdisciplinary knowledge sharing may help to identify the role of other practice team members in promoting earlier cancer presentation and diagnosis;[38] although training needs should be addressed.[37]

This study has highlighted that a 'one-size-fits-all' approach to implementing initiatives is unlikely to succeed as practitioners expressed a range of views and opinions on the benefits of different initiatives. Lack of implementation of initiatives in primary care is not always because of resistance to the initiatives themselves, but sometimes because of the sheer number across a range of priority areas that practices are expected to implement, often simultaneously. Policymakers should consider more carefully how these impact on primary care, how they can be embedded into practice systems and emphasise and exploit synergies with other disease conditions.

For there to be greater success of initiatives aimed at promoting earlier presentation, detection and referral in primary care, there needs to be further work on understanding how primary care manage risk in the face of inherent uncertainty, organisational changes and competing priorities.

For peer review only - http://bmjopen.bmj.com/site/about/guidelines.xhtml

For peer review only

ACKNOWLEDGEMENTS: Our thanks to the practices who participated in this study and to the staff of the Lancashire and South Cumbria Cancer Network who assisted us.

COMPETING INTERESTS: PD was public health lead for LSCCN and NC is part-funded by a grant from LSCCN; PD was also funded by LSCCN to undertake evaluative work.

FUNDING: This work was supported by Lancashire and South Cumbria Cancer Network (LSCCN) as part of the National Cancer Awareness and Early Detection initiative (NAEDI) funding 'cancer networks supporting primary care'.

CONTRIBUTIONS:

NC recruited participants, carried out interviews, transcribed the interviews, coded the transcripts, contributed to the development of the coding framework, and contributed to the writing of the manuscript.

GT carried out interviews, coded the transcripts, contributed to the development of the coding framework and contributed to the writing of the manuscript.

PD designed the study, collated practice engagement data, contributed to the development of the coding of the framework and contributed to the writing of the manuscript.

DATA SHARING: No further data are available

REFERENCES

1. Haward R. The Calman-Hine report: a personal retrospective on the UK's first comprehensive policy on cancer services. *The Lancet Oncology*. 2006;7(4):336-46.

For peer review only

2. Coleman MP, Foreman D, Bryant H, et al. Cancer survival in Australia, Canada, Denmark, Norway, Sweden, and the UK, 1995—2007 (the International Cancer Benchmarking Partnership): an analysis of population-based cancer registry data. *The Lancet*. 2011;377:127–38. doi:10.1016/S0140-6736(10)62231-3.
3. Daly H, Collins C. Barriers to early diagnosis of cancer in primary care: a needs assessment of GPs. *Ir Med J*. 2007; 100(10):624-26
4. Ingebrigsten SG, Scheel BI, Hart B, et al. Frequency of ‘warning signs of cancer’ in Norwegian general practice, with prospective recording of subsequent cancer. *Fam Pract*. 2013;30:153-160
5. National Institute for Clinical Excellence. Clinical Guideline 27. Referral guidelines for suspected cancer. 2005. http://www.nice.org.uk/nicemedia/live/10968/29814/29814.pdf accessed 3/1/2014.
6. Neal RD, Din NU, Hamilton W, et al. Comparison of cancer diagnostic intervals before and after implementation of NICE guidelines: analysis of data from the UK General Practice Research Database. *Br J Cancer*. 2014;110(3):584-92. doi:10.1038/bjc.2013.791. 4.
7. Mitchell ED, Rubin G, McLeod U. Understanding diagnosis of lung cancer in primary care: qualitative synthesis of significant event audit reports. *Br J Gen Pract*. 2013;63(606):e37-46. doi:10.3399/bjgp13X660760.
8. Royal College of General Practitioners. Clinical Innovation and Research Centre. National Audit of Cancer Diagnosis in Primary Care. 2011. http://www.rcgp.org.uk/news/2011/november/~/media/Files/News/National_Audit_of_Cancer_Diagnosis_in_Primary-Care.ashx. last accessed 03/01/2014.

For peer review only - http://bmjopen.bmj.com/site/about/guidelines.xhtml

9. Meechan D, Gildea C, Hollingworth L, et al. Variation in use of the 2-week referral pathway for suspected cancer: A cross-sectional analysis. *Br J Gen Pract*. 2012; 62(602):e590–97. doi:10.3399/bjgp12X654551.

10. Elliss-Brookes L, McPhail S, Ives A, et al. Routes to diagnosis for cancer - determining the patient journey using multiple routine data sets. *Br J Cancer*. 2012;107(8):1220-26. doi:10.1038/bjc.2012.408.

11. Hamilton W, Green T, Martins T et al. Evaluation of risk assessment tools for suspected cancer in general practice: a cohort study. *Br J Gen Pract*. 2013;63(606):e30-36. doi:10.3399/bjgp13X660751.

12. Emery JD, Walter FM, Gray V, et al. Diagnosing cancer in the bush: a mixed-methods study of symptom appraisal and help-seeking behaviour in people with cancer from rural Western Australia. *Fam Pract*. 2013 Jun;30(3):294-301.doi: 10.1093/fampra/cms087

13. Pedersen AF, Hansen RP, Vedsted P. Patient delay in colorectal cancer patients: associations with rectal bleeding and thoughts about cancer. *PLoS One*. 2013:Jul 22;8(7):e69700.doi: 10.1371/journal.pone.0069700

14. Marlow LA, McGregor LM, Nazroo JY, et al. Facilitators and barriers to help-seeking for breast and cervical cancer symptoms: a qualitative study with an ethnically diverse sample in London. *Psychooncology*. 2013;Dec 19.doi:10.1002/pon.3464

15. Johansen ML, Holtedahl KA, Rudebeck CE. How does the thought of cancer arise in a general practice consultation? Interviews with GPs. *Scand J Prim Health Care*. 2012;30(3):135-40. doi:10.3109/02813432.2012.688701.

16. Mitchell GK, Burridge LH, Colquist SP, et al. General Practitioners' perceptions of their role in cancer care and factors which influence this role. *Health Soc Care Community*. 2012; 20(6):607-16. doi:10.1111/j.1365-2524.2012.01075.x

For peer review only - http://bmjopen.bmj.com/site/about/guidelines.xhtml

17. Attride-Stirling J. Thematic networks: an analytic tool for qualitative research. *Qualitative Research*. 2001;1(3):385-405. doi:10.1177/146879410100100307.

18. Baughan P, Keatings J, O'Neill B. Urgent suspected cancer referrals from general practice: audit of compliance with guidelines and referral outcomes. *Br J Gen Pract*. 2011; doi:10.3399/bjgp11X606591.

19. Wallace E, Lowry J, Smith SM, et al. The epidemiology of malpractice claims in primary care: a systematic review. *BMJ Open*. 2013;3:e002929. doi:10.1136/bmjopen-2013-002929.

20. Balla J, Heneghan C, Thompson M, et al. Clinical decision making in a high-risk primary care environment: a qualitative study in the UK. *BMJ Open*. 2012;2:e000414. doi:10.1136/bmjopen-2011-000414

21. Scheel BI, Ingebrigsten SG, Thorsen T, et al. Cancer suspicion in general practice: the role of symptoms and patient characteristics, and their association with subsequent cancer. *Br J Gen Pract*. 2013;doi:10.3399/bjgp13X671614

22. Jiwa M, Gordon M, Arnet H, et al. Referring patients to specialists: a structured vignette survey of Australian and British GPs. *BMC Fam Pract*. 2008; 9:2.doi: 10.1186/1471-2296-9-2.

23. Emery J. Editorial: Assessment of cancer risk in men and women. *Br J Gen Pract*. 2013;63(606):4-5.doi:10.3399/bjgp13X660607.

24. Hamilton W. The CAPER studies: five case-control studies aimed at identifying and quantifying the risk of cancer in symptomatic primary care patients. *Br J Cancer*.2009;101: S80-S86 doi:10.1038/sj.bjc.6605396

25. André M, Andén A, Borgquist L, et al. GPs' decision-making-perceiving the patient as a person or a disease. *BMC Fam Pract*. 2012;13:38. doi:10.1186/1471-2296-13-38.

For peer review only - http://bmjopen.bmj.com/site/about/guidelines.xhtml

26. Stolper E, Van de Wiel M, Van Royen P, et al. Gut feelings as a third track in general practitioners' diagnostic reasoning. *J Gen Intern Med*. 2011;26(2):197-203. doi:10.1007/s11606-010-1524-5.

27. Zwolsman S, te Pas E, Hooft L, et al. Barriers to GPs' use of evidence-based medicine: a systematic review. *Br J Gen Pract*. 2012;62(600):e511-21. doi: 10.3399/bjgp12X652382.

28. Salah A, Hassan A. Cognitive schemes and strategies in diagnostic and therapeutic decision making: a primer for trainees. *Perspect Med Educ*. 2013; 2:321-31. doi:10.1007/s40037-013-0070-3

29. O'Riordan M, Dahinden A, Aktürk Z, et al. Dealing with uncertainty in general practice: an essential skill for the general practitioner. *Qual Prim Care*. 2011;19:175-81.

30. Brindle L, Pope C, Corner J, et al. Eliciting symptoms interpreted as normal by patients with early lung cancer. *BMJ Open*. 2012;2:e001977. doi:10.1136/bmjopen-2012-001977.

31. Round T, Steed L, Shankleman J, et al. Primary care delays in diagnosing cancer: what is causing them and what can we do about them? *J R Soc Med*. 2013;106(11):437-40. doi:10.1177/0141076813504744.

32. Walton L, McNeill R, Stevens W, et al. Patient perceptions of barriers to the early diagnosis of lung cancer and advice for health service improvement. *Fam Pract*. 2013;30:439-44 doi:10.1093/fampra/cmt001

33. Hansen RP, Vedsted P, Sokolowsk I, et al. General practitioner characteristics and delay in cancer diagnosis: a population-based cohort study. *BMC Fam Pract*. 2011; 12:100

For peer review only - http://bmjopen.bmj.com/site/about/guidelines.xhtml

34. Rogers S, Gildea C, Meechan D, et al. Access, continuity of care and consultation quality: which best predicts urgent cancer referrals from general practice? *J Public Health (Oxf).* 2014 Jan 23. [Epub ahead of print]

35. Lee J-H, Fulp W, Wells KJ, et al. Effect of patient navigation on time to diagnostic resolution among patients with colorectal cancer-related abnormalities. *J Canc Educ.* 2014;29:144-50

36. Dohan D, Schrag D. Using navigators to improve care of underserved patients. *Cancer*. 2005;104:848-55. doi:10.1002/cncr.21214.

37. Cook N, Hart A, Nuttall K, et al. A telephone survey of cancer awareness among frontline staff: informing training needs. *Br J Cancer*. 2011;105(3):340-45. doi:10.1038/bjc.

38. Kislov R, Walshe K, Harvey G. Managing boundaries in primary care service improvement: a developmental approach to communities of practice. *Implement Sci*. 2012;7:97. doi:10.1186/1748-5908-7-97.

For peer review only

Figure 1: organising themes of complexity, continuity and conflict with their associated basic themes, centred around the global theme of managing risk in primary care

For peer review only

For peer review only - http://bmjopen.bmj.com/site/about/guidelines.xhtml

Box 1: Semi-structured interview schedule

- Why do you think people with symptoms suggestive of cancer do not present, get seen or diagnosed or referred earlier?
- What do you know about the initiatives that concern the earlier presentation, diagnosis and referral of cancer symptoms?
  - Prompts around specific tools/initiatives/referral criteria
- Have you accessed any local training events concerning the identification, referral of cancer patients?
  - Prompts around specific training attended, and how learning is usually undertaken
- Overall, what is working well in terms of the implementation and use of these initiatives?
- Overall, have you experienced any/or what do you consider to be the main barriers in the implementation/access/use of these various initiatives?
- Are there any practice based issues that may affect the early identification and referral for cancer patients?
  - Prompts around staff and practice issues, communication, administration
- Overall, what do you think local practices could do to help promote and diagnose cancer symptoms?
  - Prompts around team roles and other practice issues
- Do you know about the Lancashire and South Cumbria Cancer Network and what their role is?
  - Prompts around extent of engagement, attitudes and areas for improvement
- What is your opinion concerning the forthcoming 'access to diagnostics' initiative (initiative for practices to make direct referrals to diagnostics such as x-rays, CT scans, ultrasound)?
  - Prompts around who should make the referral
- Are there any additional support mechanisms/external to the practice that need to be in place to help with promotion/diagnosis and referral?
- Any further issues or concerns you would like to raise about this work?

For peer review only - http://bmjopen.bmj.com/site/about/guidelines.xhtml

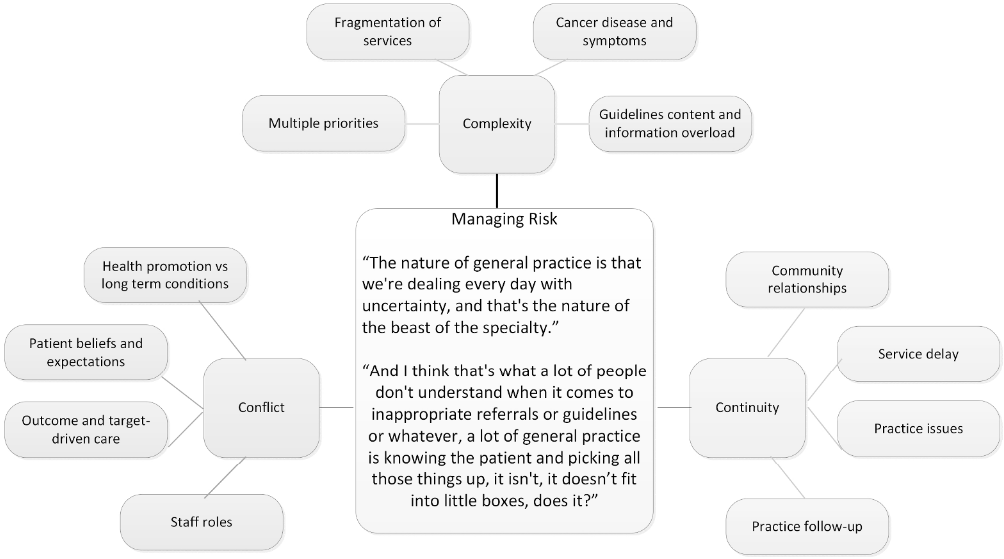

Figure 1: organising themes of complexity, continuity and conflict with their associated basic themes, centred around the global theme of managing risk in primary care
233x130mm (300 x 300 DPI)

review only

BMJ Open

Managing risk in cancer presentation, detection and referral: a qualitative study of primary care staff views

Journal:	*BMJ Open*
Manuscript ID:	bmjopen-2014-004820.R2
Article Type:	Research
Date Submitted by the Author:	19-May-2014
Complete List of Authors:	Cook, Neil; University of Central Lancashire, School of Medicine and Dentistry<br>Thomson, Gillian; University of Central Lancashire, School of Health<br>Dey, Maria; University of Central Lancashire, School of Medicine and Dentistry
**Primary Subject Heading**:	Qualitative research
Secondary Subject Heading:	Public health, General practice / Family practice
Keywords:	QUALITATIVE RESEARCH, PUBLIC HEALTH, Epidemiology < ONCOLOGY

SCHOLARONE™
Manuscripts

For peer review only

Managing risk in cancer presentation, detection and referral: a qualitative study of primary care staff views

Neil Cook, Research Assistant, School of Medicine and Dentistry, University of Central Lancashire, Preston, England*

Gillian Thomson, Senior Research Fellow, School of Health, University of Central Lancashire, Preston, England

Paola Dey, Professor of Public Health Epidemiology, School of Medicine and Dentistry, University of Central Lancashire, Preston, England

*corresponding author: Neil Cook, School of Medicine and Dentistry, Greenbank Building, University of Central Lancashire, Adelphi Street, Preston, England PR1 2HE

Email: NCook2@uclan.ac.uk Phone: 01772 893409

Key Words: primary health care, general practice, neoplasms, qualitative research, early detection of cancer.

Word count: abstract- 232, manuscript - 4,645

For peer review only

ABSTRACT

Objectives: In the UK, there have been a number of national initiatives to promote earlier detection and prompt referral of patients presenting to primary care with signs and symptoms of cancer. The aim of the study was to explore the experiences of a range of primary care staff in promoting earlier presentation, detection and referral of patients with symptoms suggestive of cancer.

Setting: Six primary care practices in North West England.

Participants: Thirty-nine primary care staff from a variety of disciplines took part in five group and four individual interviews.

Results: The global theme to emerge from the interviews was 'managing risk', which had three underpinning organising themes: 'complexity', relating to uncertainty of cancer diagnoses, service fragmentation and plethora of guidelines; 'continuity', relating to relationships between practice staff and their patients and between primary and secondary care; 'conflict' relating to policy drivers and staff role boundaries. A key concern of staff was that policymakers and those implementing cancer initiatives did not fully understand how risk was managed within primary care.

Conclusion: Primary care staff expressed a range of views and opinions on the benefits of cancer initiatives. National initiatives did not appear to wholly resolve issues in managing risk for all practitioners. Staff were concerned about the number of guidelines and priorities

For peer review only

they were expected to implement. These issues need to be considered by policymakers when developing and implementing new initiatives.

ARTICLE SUMMARY

Strengths and limitations of this study

- The study investigates the experiences of a range of primary care staff around implementing initiatives for the earlier diagnosis of cancer.
- The underlying concern in primary care is related to managing risk.
- The sample included a mix of practices with different practice characteristics and included those known to be engaged in national and regional initiatives and those who were less engaged.
- The sample was drawn from a single English region.

For peer review only

For peer review only - http://bmjopen.bmj.com/site/about/guidelines.xhtml

INTRODUCTION

Studies in the 1990s showed that UK cancer survival rates were worse than many other European countries, following which there has been two decades of concerted effort to expedite access to proven effective cancer treatments.[1] While there have been improvements in cancer survival latterly, the UK still lags behind other countries with similar health care systems, which may be partly due to later stage of disease at presentation.[2]

Both nationally and internationally, primary care is seen to have a key role in improving cancer survival by reducing delays in diagnosis through promoting earlier presentation and through earlier detection and referral of those with symptoms for further specialist assessment.[3, 4] In the UK, national campaigns extol those with symptoms to see their general practitioner earlier; there are national referral guidelines for suspected cancer and a national system for urgent referral from primary to secondary care (two-week waiting-time initiative).[5] While these appear to have contributed to improved survival, evidence also suggests that there may be further room for improvement:[6] there is practice variation in the use of the 'two-week' initiative and some patients are seen several times in primary care before referral.[7-9] A significant proportion of patients also present through emergency routes and have poorer survival.[10]

To further support primary care in the UK, resources were developed and/or disseminated by the Department of Health's National Awareness and Early Diagnosis Initiative (NAEDI), established in 2008, and the Royal College of General Practitioners (RCGP) including: audit and significant events analysis tools,[7, 8] general practitioner (GP) level cancer profile data, safety netting recommendations and risk assessment tools.[11] Key initiatives have mainly

For peer review only - http://bmjopen.bmj.com/site/about/guidelines.xhtml

focused on GPs, but other members of the primary care team may have key roles: an area which has been largely unexplored.

A number of international studies have been undertaken in attempts to understand the reasons behind delay in cancer diagnosis. Qualitative studies have mostly focussed on patient perspectives.[12-14] Only a few have explored primary care experiences and these have been mainly limited to decision-making processes or referral pathways [11, 15, 16] and from the perspective of the general practitioner.[3, 11, 15, 16] In order to understand how a range of initiatives across the patient pathway in primary care could be more effective and the role of other members of the practice team, we explored the experiences of a range of primary care staff in supporting earlier presentation, detection and referral of those with symptoms suggestive of cancer. The study was undertaken in one region of England, which at that time was covered by the Lancashire and South Cumbria Cancer Network (LSCCN).

METHODS

This was a qualitative descriptive study utilising both individual and group-based interviews. It aimed to recruit staff from six practices who were differentially engaged with the national awareness and early diagnosis of cancer agenda. GP practices within LSSCN were stratified into one of three groups at the end of June 2012. High engagers had participated in at least one of the following: RCGP cancer audit, attendance at a course on early diagnosis or face-to-face meetings to discuss GP cancer profiles and action planning. Medium engagers had attended at least one regional meeting about cancer awareness. Low engagers were not

For peer review only

known to have engaged in any initiatives. Of 254 practices in the geographical area, there were 51 high engagers, 69 medium engagers and 134 low engagers.

Within each group, a random sample of 10 practices were sent a letter about the study, followed up with a phone call from the research team one week later. The initial aim was to recruit at least two practices in each stratum. Due to low uptake within the medium and low engager strata, these categories were merged and a random sample of a further 15 practices sent letters. Practices were offered a choice of either group or individual interviews as it was recognised that time constraints prevent some practice staff from taking part in group interviews and some may feel uncomfortable discussing the issues with colleagues. Five practices agreed to a group interview; with one group interview undertaken in each practice. Individual interviews were the preferred method in only one practice: with four interviews undertaken in this setting.

The semi-structured, audio-recorded interviews, which were attended by two researchers, occurred between September and October 2012. We recruited three practices in the high stratum and three practices in the merged medium and low stratum (a mix of low and medium engagers). The topic guide is outlined in Box 1. Thirty-nine participants took part in the study; 35 took part in one of the five group interviews and four took part in individual interviews. Job roles included GP (n=9), receptionist (n=7), nurse (n=6), manager (n=6), secretary (n=5), health care assistant (n=3), medical student (n=2) and phlebotomist (n=1). In each practice, GPs, other clinical staff and administrative staff were involved in the interviews. Practice characteristics are shown in table 1.

For peer review only - http://bmjopen.bmj.com/site/about/guidelines.xhtml

Table 1: Practice and interview characteristics

Practice characteristic	No. of practices
No of partners	
4 or more	3
3 or less	3
Deprivation Quintile*	
5 or 4 (more deprived)	3
2 or 3	3
1 (most affluent)	0
Population Density**	
<15%	2
15 to 39%	2
>=40%	2

*based on index of multiple deprivation of practice location 2010 (source: Department for Communities and Local Government, Indices of Deprivation 2010)
** Person per hectare based on practice location (source: ONS Neighbourhood Statistics)

Further detail at practice level is not presented due to the level of confidentiality agreed with our participants.

The interviews took between 38 and 67 minutes to complete, and between four and eleven staff took part in each practice. All transcribed data were entered into NVivo 10 and analysed using a Thematic Network Analysis approach.[17] This involved an iterative and cyclical process of reading and analysis to identify basic, organising and global themes within the dataset. Analysis was undertaken by two authors (GT and NC) independently on six transcripts initially, followed by an in-depth discussion and consensual validation of key themes. A further cycle of independent and collaborative analysis was then undertaken on a further sub-set of transcripts to ensure rigour and authenticity of the themes generated. All thematic decisions were discussed with the third author (PD). The study was approved by the

For peer review only - http://bmjopen.bmj.com/site/about/guidelines.xhtml

University of Central Lancashire 'STEMH' ethics committee and individual written informed consent was obtained from all participants.

RESULTS

Overall, practice staff were well aware of the two-week waiting-time initiative and had good knowledge about the national 'Be Clear on Cancer' cancer awareness public campaigns, but had less awareness about other initiatives specifically targeted at primary care. The key global theme to emerge from the interviews related to 'managing risk' within primary care:

> *"It's quite a tricky, nebulous area* […]. *The nature of general practice is that we're dealing every day with uncertainty…"* (Interview 6, Participant 3)

A key concern of staff was that policymakers and those implementing cancer initiatives did not fully understand how risk was managed within primary care. Cancer was only one priority and there was an abundance of initiatives for a variety of conditions which primary care staff were expected to implement. Three underpinning organising themes (and associated basic themes) of 'complexity', 'continuity' and 'conflict' highlighted the tensions and difficulties that primary care face in managing the risk of early detection and referral for cancer symptoms whilst dealing with complex symptoms and care systems, patient-led factors and target-focused care. An overview of the organising and basic themes is presented in figure 1. These themes are described and discussed, contextualised by participant quotes below.

Complexity

For peer review only - http://bmjopen.bmj.com/site/about/guidelines.xhtml

This theme highlighted the complexity of managing risk in early cancer diagnosis because of external factors including cancer symptom differentiation and the restrictions imposed by referral criteria; the multitude of services and professionals involved in diagnostic and assessment services and the plethora of policies and initiatives targeted at primary care practice.

Cancer disease and symptoms: Cancer diagnosis was an important priority area in primary care but diagnosis presented complex challenges. Cancer was a rare diagnosis in primary care although symptoms associated with cancer were common.

> *"We get lots of sore throats, and yet we get one tonsillar cancer every three* [years] *so sorting out the wheat from the chaff is a real challenge."* (Interview 3, Participant 3)

These complexities of diagnosis were compounded by what were considered rigid referral criteria, based on disease prevalence amongst those with symptoms, which led to cases being '*bounced back'* if they failed to meet diagnostic criteria. In attempts to manage what they considered was a risk to patients, some participants said, on occasion, they had to '*fudge'*, '*embellish'* or '*bend-the-rules'* to ensure assessment of patients for whom they had concerns:

> *"Even though it says on the form, "don't fill out this form unless they tick any boxes" but you find a box to tick, and usually for very good reason. And I think you'd only go slightly over egging the presentation if you were pretty sure there was something there, something going on."* (Interview 4, Participant 4)

For peer review only - http://bmjopen.bmj.com/site/about/guidelines.xhtml

Fragmentation and access to diagnostic services: Primary care staff expressed difficulties accessing diagnostic services due to on-going service re-configurations and the involvement of multiple agencies. This led to fragmentation in terms of staff not always knowing who, or to which services, referrals could be made.

There were also some concerns about fragmented relationships between primary and secondary care with several participants feeling frustrated by restricted access to diagnostics for certain conditions. Some participants considered this was due to *'empire building'* by professionals justifying and *'preserving'* their service by retaining ownership of who was qualified to make referrals:

> *"I can send people for a CT scan if I have a concern about them, for some conditions, but not others. Well why not? I'm the one who's initiating the referral in the first place. You trust me to initiate the referral to pick the patient and prepare them so that you can come along and just arrange the scan and look at it."* (Interview 4, Participant 3)

Whilst some GPs wanted more direct access to diagnostics, this was not universal, and others highlighted the need for *'training'* prior to referrals being made.

Guidelines content and information overload: Participants referred to the usefulness of guidelines to help symptom differentiation and manage risk. Knowledge was felt to be experientially derived and referral guidelines only considered useful for less experienced doctors. Risk assessment tools were occasionally considered unnecessarily complex when

For peer review only - http://bmjopen.bmj.com/site/about/guidelines.xhtml

patients had ‘red flag’ symptoms and, as previously mentioned, sometimes restrictive for use in primary care:

> *“I mean, you see, from a GPs point of view, if somebody comes in who is fifty-five years of age, and passes blood in his stool, you need to exclude a cancer. Now, I don’t want to know how many percentage of those people pass blood in the motion will have cancer, or how many will have piles, or inflammatory bowel disease or what have you.”* (Interview 4, Participant 3)

Concerns were expressed about the number of guidelines available for different conditions and inconsistencies between different sources:

> *“And that's the problem ‘cause last year there was a big campaign, “if you have a cough for more than three weeks you need to go and see a doctor”, but NICE guidelines is six weeks.*” (Interview 6, Participant 4)

Participants described how the *‘tsunami’* of new guidelines, care plans and initiatives meant it was difficult to keep up-to-date:

> *“And I thought ‘that’s probably a squamous cell carcinoma. That needs a two-week wait referral’. Then, I actually checked the guidelines, the guidelines say that “it’s got to be over one centimetre and you've got to wait more than eight weeks really for it to grow” etcetera, etcetera* […] *and those things will change on a regular basis. Now, how do you expect an entire network of primary care physicians to stay current with all those guidelines and apply them religiously?”* (Interview 2, Participant 3).

For peer review only - http://bmjopen.bmj.com/site/about/guidelines.xhtml

For peer review only

Continuity

Practice staff expressed concern that a lack of continuity increased the risk of missing diagnoses and/or supporting the patient through a difficult period. This was highlighted through discussions about relationships between patients and clinicians; delays in information sharing across the primary and secondary care interface and patient follow-up after initial consultation.

Patient-practice relationships: Continuity-of-carer could not always be achieved in practice, even though it was felt that patients preferred it and its absence was thought to possibly contribute to diagnostic delay:

> *"People have been hopping around from one clinician to another and that, and you don't see the evolution of the story until it hits you in the face. Patients book on the day, they don't necessarily get in the person they normally see, they get in with the available, so that can disjoint things."* (Interview 3, Participant 4)

Staff felt that smaller, rural practices had closer relationships with their patients and their community, which would lead to earlier consultation. However, others felt awareness of cancer deaths was heightened in close-knit communities, which could reinforce negative views towards cancer and cancer survivorship amongst the local population and delay access to services.

Primary care and secondary care interface: Expedited assessment following GP referral of patients through the two-week wait initiative was perceived to work well by practice staff,

For peer review only - http://bmjopen.bmj.com/site/about/guidelines.xhtml

but delays further on in the assessment process, and outside of primary care control, were felt to put patients at risk again and exacerbated their concerns:

> *"And, well, it's very difficult from our point of view, 'cause we're saying, "look, you know this may be nothing serious, however, I want to refer you on the system, you'll get an appointment in two weeks", and they do get seen, but then there's a massive delay, so, you know, then they're kinda all heightened up because they're thinking, "{whispered} the doctor thinks I've got cancer" and then nothing happens for ages, and it's really hard for the patients."* (Interview 3, Participant 5)

Practice-based follow-up: Patients themselves were also considered to cause delays if they failed to attend. Practices debated the extent to which they should chase these patients up and the risks if they did not:

> *"In the back of their mind they know they should, but if they face the fact that they're going back, are they are going to be faced with something else? You also have to be responsible for the fact that if that doesn't stop, you must persist, and accept the fact that, if you don't, then you could, somewhere down the line end up with something so serious that it's only going to be palliative."* (Interview 2, Participant 1)

Conflict

Within this theme, managing risk was related to primary care focus on targets; conflicting opinions about the role of non-clinical staff and about the worries and tensions generated by cancer awareness campaigns.

For peer review only - http://bmjopen.bmj.com/site/about/guidelines.xhtml

Prevention versus target driven care: Primary care staff considered cancer a public health priority but some felt that there was a tension between adopting preventative approaches in the practice such as promoting cancer awareness and early presentation and the way that primary care is currently funded through achieving specific targets mainly relating to the management of long-term conditions (Quality and Outcomes Framework):

> *"We're not as good with public health, with local public health initiatives, as maybe we should be necessarily. It's just time to do things, and those things aren't required of our core business, and when you have a very detailed contract that tells you what you will be paid for doing"* (Interview 2, Participant 3)

Staff roles: There were conflicting views around the role of other practice staff in managing the risk of cancer detection and awareness, in particular with regard to reception staff. Some staff viewed the reception role as a health advisor, whereas others perceived their role to be purely administrative. Additionally, there were reservations about the ability of reception staff to relay cancer messages and how the public might feel about receiving health information from them:

> *"You're passing clinical responsibility to people who are non-clinical. It's alright for patients to speak to them, and that's fine, but, I'm not gonna use the receptionists as a source of information* […]. *I think anything clinical should be passed to the doctor full stop. I don't think there is any other role for the receptionist."* (Interview 4, Participant 3)

For peer review only - http://bmjopen.bmj.com/site/about/guidelines.xhtml

For peer review only

There were also concerns about potential litigation issues if reception staff were to give advice and how this could impact on the practice:

> *"I think it's also worth mentioning at this point, because of our roles, how they are, we get a lot of patients not happy about us supplying information, because we're not allowed to diagnose, obviously for obvious reasons, but if you was to imply something, that could come back on us twice as hard because you'd implied something that could be wrong, and therefore it is now your fault."* (Interview 1, Participant 1)

A further issue associated with staff roles in helping to manage risk was the identified benefits of having a chain of communication between all practice members. For example, receptionists being able to *'raise issues'* with the doctor if they know someone is coming in who has symptoms the doctor should be aware of but the patient *'may not say anything'*. This can also work the other way:

> *"Patients that I worry about, I will leave messages. I'll mention to reception or mention to people that I want to follow them up."* (Interview 6, Participant 3)

Others felt that the focus on the long-term conditions might provide opportunities for practice nurses to be more involved as they were often seeing patients who might be at higher risk of cancer because of their age, disease condition or lifestyle behaviours:

> *"Chronic disease, yeah, so diabetic, high blood pressure, chronic kidney disease, asthma, most patients will come and see the practice nurses for routine bloods, blood*

For peer review only - http://bmjopen.bmj.com/site/about/guidelines.xhtml

pressure, weight, everything like that. So often, GPs don't always see them, I mean they do the medication reviews, but, we flag to them anything that we're worried about." (Interview 2, Participant 4)

Some could also see a role for members of the wider practice team:

"We'd say 'well who are the district nurses already going out to?' You know, you can work with your local pharmacists." (Interview 2, Participant 2)

Cancer awareness: Although cancer fear was commonly acknowledged, staff were divided as to whether patients were afraid to consult the GP with cancer symptoms. Some felt that unhealthy lifestyle choices contributed to patient reluctance to present with cancer symptoms:

"The cancers where they feel that they may have contributed to it, like smoking, they tend to ignore because they don't want to be told that it may be their fault in a certain way, and they don't want to give up the lifestyle." (Interview 6, Participant 4)

Cancer awareness campaigns were felt to be important, even though those who presented to the practice following the campaign were more likely to be those at least risk:

"It's the people who never come to the doctors that you want to hit, not the people that were already coming in anyway, and they tend to be ones that see those adverts. But then if you can get one person who wouldn't normally come in and you catch them, then it's better." (Interview 5, Participant 1)

For peer review only - http://bmjopen.bmj.com/site/about/guidelines.xhtml

Campaigns were also felt to create extra work for all practice members. There were complaints that cancer awareness campaigns tend to run without consideration of other local or national campaigns which may be running simultaneously which increased the risk that practice capacity to safely respond to patients was compromised:

> *"It's like it's alright saying putting in place to get them in, but it's the sheer* volume*, isn't it? You can only cope with so much can't you?"* (Interview 5, Participant 1)

DISCUSSION

This study provides insights into the experiences of primary care staff who manage patients with symptoms associated with cancer. The overarching theme that emerged was the need to manage risk so that cancer patients had a timely diagnosis and were assessed appropriately. The associated subthemes of complexity, continuity and conflict highlighted the tensions and difficulties faced by staff when attempting to manage these risks in modern practice.

The study was small, descriptive and exploratory. However, it covered a diverse geographical area and included practices with varying levels of engagement with awareness and early diagnosis initiatives. We undertook stratified sampling of the practices to ensure we had a balance of perspectives so as to better inform policymakers of the possible barriers and drivers to the uptake of initiatives. The stratification was based on known engagement with a range of national and regional initiatives to promote earlier presentation, detection and referral of patients with symptoms suggestive of cancer available to the practices at that time.

For peer review only - http://bmjopen.bmj.com/site/about/guidelines.xhtml

The findings of our study suggest that national initiatives did not appear to wholly resolve issues around managing risk for all practitioners. Rather, in some cases, these initiatives were felt to introduce inherent risks which staff had to find ways to overcome. For example, national cancer awareness campaigns were felt to be very important to encourage patients who would otherwise delay diagnosis. However, as these campaigns appeared to increase consultation rates from those at lesser risk, this was perceived to place additional burden on the practice.

There were also concerns amongst our participants about practice-based initiatives. The International Cancer Benchmarking Partnership observed lower survival rates in Denmark and the UK compared to other countries in their study, and raised concerns about the role of primary care gatekeeping in delayed diagnosis.[2] This observation appears to be supported by a wider study of European countries which also demonstrated lower cancer survival in those with primary care based gatekeeper systems.[18] Primary care gatekeeping may reduce the burden on specialist care but may also contribute to diagnostic delay through the restriction of access to these services. In the UK, national referral guidelines and the two-week waiting-time initiative should help alleviate this problem for those with symptoms associated with cancer. Participants in our study considered that timely diagnosis was as an essential part of their role in cancer care. An Australian study had a similar finding,[16] but highlighted that the resources spent gaining timely access to specialist opinion were a major issue for their practitioners. In our study this was universally considered less of a problem because of the two-week waiting-time initiative, but concerns were expressed that lack of direct access to diagnostic investigations and poor communication between primary and secondary care put patients at risk of extended delays. Similar concerns were reported in a study undertaken in Ireland.[3] Some participants in our study felt that the referral criteria for

For peer review only - http://bmjopen.bmj.com/site/about/guidelines.xhtml

the two-week waiting-time initiative were too restrictive. This led to practitioners, on occasion, subverting the referral system to ensure that patients they considered were at risk, but who did not fit the referral criteria, could be assessed in a timely manner. Such concerns are not unfounded; one study has shown that 8% of patients felt by GPs to have cancer, but who did not have symptoms which fit referral criteria for cancer, were subsequently diagnosed with cancer.[19] This may be because the presenting signs and symptoms had a lower predictive value for cancer than those included in the guidelines. Nevertheless, there is high compliance with guidelines and some limited evidence that referral guidelines contribute, in part, to diagnostic delay reduction.[6, 19]

Diagnostic errors leading to primary care malpractice claims are common.[20] Researchers suggest that although GPs are more likely to correctly diagnose patients with cancer than miss cases, there are a disproportionate number of deaths amongst the latter.[21,22] Diagnostic complexity is compounded by the frequency of consultations for symptoms associated with cancer,[22, 23] a fear highlighted by practitioners in this study. A study in Norway, which followed up patients presenting with cancer warning signs to their GP, suggests that patients with cancer may be missed if multiple warning signs and symptoms are not considered.[22] Retrospective studies of practice-based data have identified combinations of warning signs and symptoms which may be associated with increasing likelihood of cancer.[24-25] These have been used to inform risk assessment tools to aid decision-making, with algorithms and probabilities of risk based on demographic characteristics, lifestyle factors, symptoms and/or attendance frequency.[11] Some have been disseminated nationally, and there is evidence from both quantitative and qualitative studies that they affect GPs' decisions to refer.[11] However, in our study, some participants had similar concerns about these tools as they did about guidelines. A recent analysis of

For peer review only - http://bmjopen.bmj.com/site/about/guidelines.xhtml

significant event audits in lung cancer highlighted how cancer can mimic other diagnoses and have atypical presentation.[7] Guidelines and risk assessment tools are analytical tools, and although statistical probabilities are based on uncertainty, some participants in our study felt they may introduce a level of certainty which fails to encapsulate practitioners' tacit concerns about patients. Previous work in primary care decision-making has emphasised the analytical over the experiential, but recent research , including in cancer diagnosis, suggests that experiential knowledge may have a role and may be more responsive to the patient as a person.[15, 26-28] Others have found that better diagnostic decisions about urgent referrals appear to be made by older doctors [23] and, as commented on by some of our participants, that guidelines and risk assessment tools may be more useful for newer practitioners who have yet to develop problem solving strategies.[29]

It has also been observed that, unlike in acute care, decision-making in primary care partly involves an understanding of the patient context and perspective.[30] Our study participants highlighted concerns that lack of continuity of carer may lead to diagnostic delays. Relational continuity was felt to be a particular problem in larger practices and urban settings. Other studies suggest that fragmentation of primary care and shorter consultation times has led to a lack of continuity-of-care or carer, which may hinder early diagnosis as cancer presentation can be complex in those with co-morbidities.[16, 31, 32] In a qualitative study of lung cancer patients in New Zealand, patients felt that not always seeing the same GP could lead to delays in diagnosis due to poor follow up.[33] In a study in Denmark, the authors suggest that perceived lack of accessibility and doctor-patient relationship were associated with patient delay in seeking advice about cancer symptoms.[34] Others have found that confidence and trust in a doctor were more important predictors of cancer detection than ease of access and choice of preferred doctor.[35]

For peer review only - http://bmjopen.bmj.com/site/about/guidelines.xhtml

In our study, participants highlighted how other practice team members, such as nurses who are in more regular contact with patients with long-term conditions, could raise symptom awareness or be more alert to symptoms suggestive of cancer. In the US and Canada, patient navigators are being used to support patients through the complex systems found in cancer management and care.[36] It has also been suggested that these roles could be extended to support patients during the diagnostic, referral and assessment processes and ensure appropriate follow up of investigations.[37] In our study, some staff felt that receptionists could act as navigators in terms of directing patients with symptoms to see the GP. Low levels of knowledge about some cancer symptoms has been demonstrated in non-medical staff.[38] However, our study highlighted that professional boundaries and concerns about litigation might impede such initiatives. Disclosures or discussions of diagnostic issues within a public reception location also raised ethical concerns. Research within primary care suggests that facilitation of communities of practice and interdisciplinary knowledge sharing may help to identify the role of other practice team members in promoting earlier cancer presentation and diagnosis;[39] although training needs should be addressed.[38]

This study has highlighted that a ‘one-size-fits-all’ approach to implementing initiatives is unlikely to succeed as practitioners expressed a range of views and opinions on the benefits of different initiatives. Lack of implementation of initiatives in primary care is not always because of resistance to the initiatives themselves, but sometimes because of the sheer number across a range of priority areas that practices are expected to implement, often simultaneously. Policymakers should consider more carefully how these impact on primary care, how they can be embedded into practice systems and emphasise and exploit synergies with other disease conditions.

For peer review only - http://bmjopen.bmj.com/site/about/guidelines.xhtml

For there to be greater success of initiatives aimed at promoting earlier presentation, detection and referral in primary care, there needs to be further work on understanding how primary care manage risk in the face of inherent uncertainty, organisational changes and competing priorities.

ACKNOWLEDGEMENTS: Our thanks to the practices who participated in this study and to the staff of the Lancashire and South Cumbria Cancer Network who assisted us.

COMPETING INTERESTS: PD was public health lead for LSCCN and NC is part-funded by a grant from LSCCN; PD was also funded by LSCCN to undertake evaluative work.

FUNDING: This work was supported by Lancashire and South Cumbria Cancer Network (LSCCN) as part of the National Cancer Awareness and Early Detection initiative (NAEDI) funding 'cancer networks supporting primary care'.

CONTRIBUTIONS:

NC recruited participants, carried out interviews, transcribed the interviews, coded the transcripts, contributed to the development of the coding framework, and contributed to the writing of the manuscript.

GT carried out interviews, coded the transcripts, contributed to the development of the coding framework and contributed to the writing of the manuscript.

PD designed the study, collated practice engagement data, contributed to the development of the coding of the framework and contributed to the writing of the manuscript.

For peer review only - http://bmjopen.bmj.com/site/about/guidelines.xhtml

DATA SHARING: No further data are available

REFERENCES

1. Haward R. The Calman-Hine report: a personal retrospective on the UK's first comprehensive policy on cancer services. *The Lancet Oncology*. 2006;7(4):336-46.
2. Coleman MP, Foreman D, Bryant H, et al. Cancer survival in Australia, Canada, Denmark, Norway, Sweden, and the UK, 1995—2007 (the International Cancer Benchmarking Partnership): an analysis of population-based cancer registry data. *The Lancet*. 2011;377:127–38. doi:10.1016/S0140-6736(10)62231-3.
3. Daly H, Collins C. Barriers to early diagnosis of cancer in primary care: a needs assessment of GPs. *Ir Med J*. 2007; 100(10):624-26
4. Ingebrigsten SG, Scheel BI, Hart B, et al. Frequency of 'warning signs of cancer' in Norwegian general practice, with prospective recording of subsequent cancer. *Fam Pract*. 2013;30:153-160
5. National Institute for Clinical Excellence. Clinical Guideline 27. Referral guidelines for suspected cancer. 2005. http://www.nice.org.uk/nicemedia/live/10968/29814/29814.pdf accessed 3/1/2014.
6. Neal RD, Din NU, Hamilton W, et al. Comparison of cancer diagnostic intervals before and after implementation of NICE guidelines: analysis of data from the UK General Practice Research Database. *Br J Cancer*. 2014;110(3):584-92. doi:10.1038/bjc.2013.791. 4.
7. Mitchell ED, Rubin G, MacLeod U. Understanding diagnosis of lung cancer in primary care: qualitative synthesis of significant event audit reports. *Br J Gen Pract*. 2013;63(606):e37-46. doi:10.3399/bjgp13X660760.

For peer review only - http://bmjopen.bmj.com/site/about/guidelines.xhtml

8. Royal College of General Practitioners. Clinical Innovation and Research Centre. National Audit of Cancer Diagnosis in Primary Care. 2011. http://www.rcgp.org.uk/news/2011/november/~/media/Files/News/National_Audit_of_Cancer_Diagnosis_in_Primary-Care.ashx. accessed 03/01/2014.
9. Meechan D, Gildea C, Hollingworth L, et al. Variation in use of the 2-week referral pathway for suspected cancer: A cross-sectional analysis. *Br J Gen Pract*. 2012; 62(602):e590–97. doi:10.3399/bjgp12X654551.
10. Elliss-Brookes L, McPhail S, Ives A, et al. Routes to diagnosis for cancer - determining the patient journey using multiple routine data sets. *Br J Cancer*. 2012;107(8):1220-26. doi:10.1038/bjc.2012.408.
11. Hamilton W, Green T, Martins T et al. Evaluation of risk assessment tools for suspected cancer in general practice: a cohort study. *Br J Gen Pract*. 2013;63(606):e30-36. doi:10.3399/bjgp13X660751.
12. Emery JD, Walter FM, Gray V, et al. Diagnosing cancer in the bush: a mixed-methods study of symptom appraisal and help-seeking behaviour in people with cancer from rural Western Australia. *Fam Pract*. 2013 Jun;30(3):294-301.doi: 10.1093/fampra/cms087
13. Pedersen AF, Hansen RP, Vedsted P. Patient delay in colorectal cancer patients: associations with rectal bleeding and thoughts about cancer. *PLoS One*. 2013:Jul 22;8(7):e69700.doi: 10.1371/journal.pone.0069700
14. Marlow LA, McGregor LM, Nazroo JY, et al. Facilitators and barriers to help-seeking for breast and cervical cancer symptoms: a qualitative study with an ethnically diverse sample in London. *Psychooncology*. 2013;Dec 19.doi:10.1002/pon.3464

For peer review only - http://bmjopen.bmj.com/site/about/guidelines.xhtml

15. Johansen ML, Holtedahl KA, Rudebeck CE. How does the thought of cancer arise in a general practice consultation? Interviews with GPs. *Scand J Prim Health Care*. 2012;30(3):135-40. doi:10.3109/02813432.2012.688701.

16. Mitchell GK, Burridge LH, Colquist SP, et al. General Practitioners' perceptions of their role in cancer care and factors which influence this role. *Health Soc Care Community*. 2012; 20(6):607-16. doi:10.1111/j.1365-2524.2012.01075.x

17. Attride-Stirling J. Thematic networks: an analytic tool for qualitative research. *Qualitative Research*. 2001;1(3):385-405. doi:10.1177/146879410100100307.

18. Vedsted P and Olesen F. Are the serious problems in cancer survival partly rooted in gatekeeper principles? An ecologic study. *Br J Gen Pract*. 2011;DOI:10.3399/bjgp11X588484.

19. Baughan P, Keatings J, O'Neill B. Urgent suspected cancer referrals from general practice: audit of compliance with guidelines and referral outcomes. *Br J Gen Pract*. 2011; doi:10.3399/bjgp11X606591.

20. Wallace E, Lowry J, Smith SM, et al. The epidemiology of malpractice claims in primary care: a systematic review. *BMJ Open*. 2013;3:e002929. doi:10.1136/bmjopen-2013-002929.

21. Wallis K, Dovey S. No fault compensation for treatment injury in New Zealand: identifying threats to patient safety in primary care. BMJ Quality Safety. 2011;20(7):587-91. doi: 10.1136/bmjqs.2010.0476906.

22. Scheel BI, Ingebrigtsen SG, Thorsen T, et al. Cancer suspicion in general practice: the role of symptoms and patient characteristics, and their association with subsequent cancer. *Br J Gen Pract*. 2013;doi:10.3399/bjgp13X671614

For peer review only - http://bmjopen.bmj.com/site/about/guidelines.xhtml

23. Jiwa M, Gordon M, Arnet H, et al. Referring patients to specialists: a structured vignette survey of Australian and British GPs. *BMC Fam Pract*. 2008; 9:2.doi: 10.1186/1471-2296-9-2.

24. Emery J. Editorial: Assessment of cancer risk in men and women. *Br J Gen Pract*. 2013;63(606):4-5.doi:10.3399/bjgp13X660607.

25. Hamilton W. The CAPER studies: five case-control studies aimed at identifying and quantifying the risk of cancer in symptomatic primary care patients. *Br J Cancer*.2009;101: S80-S86 doi:10.1038/sj.bjc.6605396

26. André M, Andén A, Borgquist L, et al. GPs' decision-making-perceiving the patient as a person or a disease. *BMC Fam Pract*. 2012;13:38. doi:10.1186/1471-2296-13-38.

27. Stolper E, Van de Wiel M, Van Royen P, et al. Gut feelings as a third track in general practitioners' diagnostic reasoning. *J Gen Intern Med*. 2011;26(2):197-203. doi:10.1007/s11606-010-1524-5.

28. Zwolsman S, te Pas E, Hooft L, et al. Barriers to GPs' use of evidence-based medicine: a systematic review. *Br J Gen Pract*. 2012;62(600):e511-21. doi: 10.3399/bjgp12X652382.

29. Salah A, Hassan A. Cognitive schemes and strategies in diagnostic and therapeutic decision making: a primer for trainees. *Perspect Med Educ.* 2013; 2:321-31. doi:10.1007/s40037-013-0070-3

30. O'Riordan M, Dahinden A, Aktürk Z, et al. Dealing with uncertainty in general practice: an essential skill for the general practitioner. *Qual Prim Care*. 2011;19:175-81.

31. Brindle L, Pope C, Corner J, et al. Eliciting symptoms interpreted as normal by patients with early lung cancer. *BMJ Open*. 2012;2:e001977. doi:10.1136/bmjopen-2012-001977.

For peer review

For peer review only - http://bmjopen.bmj.com/site/about/guidelines.xhtml

32. Round T, Steed L, Shankleman J, et al. Primary care delays in diagnosing cancer: what is causing them and what can we do about them? *J R Soc Med*. 2013;106(11):437-40. doi:10.1177/0141076813504744.

33. Walton L, McNeill R, Stevens W, et al. Patient perceptions of barriers to the early diagnosis of lung cancer and advice for health service improvement. *Fam Pract*. 2013;30:439-44 doi:10.1093/fampra/cmt001

34. Hansen RP, Vedsted P, Sokolowsk I, et al. General practitioner characteristics and delay in cancer diagnosis: a population-based cohort study. *BMC Fam Pract*. 2011; 12:100

35. Rogers S, Gildea C, Meechan D, et al. Access, continuity of care and consultation quality: which best predicts urgent cancer referrals from general practice? *J Public Health (Oxf).* 2014 Jan 23. [Epub ahead of print]

36. Dohan D, Schrag D. Using navigators to improve care of underserved patients. *Cancer*. 2005;104:848-55. doi:10.1002/cncr.21214.

37. Lee J-H, Fulp W, Wells KJ, et al. Effect of patient navigation on time to diagnostic resolution among patients with colorectal cancer-related abnormalities. *J Canc Educ.* 2014;29:144-50

38. Cook N, Hart A, Nuttall K, et al. A telephone survey of cancer awareness among frontline staff: informing training needs. *Br J Cancer*. 2011;105(3):340-45. doi:10.1038/bjc.

39. Kislov R, Walshe K, Harvey G. Managing boundaries in primary care service improvement: a developmental approach to communities of practice. *Implement Sci.* 2012;7:97. doi:10.1186/1748-5908-7-97.

For peer review only - http://bmjopen.bmj.com/site/about/guidelines.xhtml

Figure 1: organising themes of complexity, continuity and conflict with their associated basic themes, centred around the global theme of managing risk in primary care

For peer review only

For peer review only - http://bmjopen.bmj.com/site/about/guidelines.xhtml

Box 1: Semi-structured interview schedule

- Why do you think people with symptoms suggestive of cancer do not present, get seen or diagnosed or referred earlier?
- What do you know about the initiatives that concern the earlier presentation, diagnosis and referral of cancer symptoms?
  - Prompts around specific tools/initiatives/referral criteria
- Have you accessed any local training events concerning the identification, referral of cancer patients?
  - Prompts around specific training attended, and how learning is usually undertaken
- Overall, what is working well in terms of the implementation and use of these initiatives?
- Overall, have you experienced any/or what do you consider to be the main barriers in the implementation/access/use of these various initiatives?
- Are there any practice based issues that may affect the early identification and referral for cancer patients?
  - Prompts around staff and practice issues, communication, administration
- Overall, what do you think local practices could do to help promote and diagnose cancer symptoms?
  - Prompts around team roles and other practice issues
- Do you know about the Lancashire and South Cumbria Cancer Network and what their role is?
  - Prompts around extent of engagement, attitudes and areas for improvement
- What is your opinion concerning the forthcoming ‘access to diagnostics’ initiative (initiative for practices to make direct referrals to diagnostics such as x-rays, CT scans, ultrasound)?
  - Prompts around who should make the referral
- Are there any additional support mechanisms/external to the practice that need to be in place to help with promotion/diagnosis and referral?
- Any further issues or concerns you would like to raise about this work?

For peer review only - http://bmjopen.bmj.com/site/about/guidelines.xhtml

Managing risk in cancer presentation, detection and referral: a qualitative study of primary care staff views

Neil Cook, Research Assistant, School of Medicine and Dentistry, University of Central Lancashire, Preston, England*

Gillian Thomson, Senior Research Fellow, School of Health, University of Central Lancashire, Preston, England

Paola Dey, Professor of Public Health Epidemiology, School of Medicine and Dentistry, University of Central Lancashire, Preston, England

*corresponding author: Neil Cook, School of Medicine and Dentistry, Greenbank Building, University of Central Lancashire, Adelphi Street, Preston, England PR1 2HE

Email: NCook2@uclan.ac.uk Phone: 01772 893409

Key Words: primary health care, general practice, neoplasms, qualitative research, early detection of cancer.

Word count: abstract- 232, manuscript - ~~4,504~~4,645

For peer review only

For peer review only - http://bmjopen.bmj.com/site/about/guidelines.xhtml

ABSTRACT

Objectives: In the UK, there have been a number of national initiatives to promote earlier detection and prompt referral of patients presenting to primary care with signs and symptoms of cancer. The aim of the study was to explore the experiences of a range of primary care staff in promoting earlier presentation, detection and referral of patients with symptoms suggestive of cancer.

Setting: Six primary care practices in North West England.

Participants: Thirty-nine primary care staff from a variety of disciplines took part in five group and four individual interviews.

Results: The global theme to emerge from the interviews was 'managing risk', which had three underpinning organising themes: 'complexity', relating to uncertainty of cancer diagnoses, service fragmentation and plethora of guidelines; 'continuity', relating to relationships between practice staff and their patients and between primary and secondary care; 'conflict' relating to policy drivers and staff role boundaries. A key concern of staff was that policymakers and those implementing cancer initiatives did not fully understand how risk was managed within primary care.

Conclusion: Primary care staff expressed a range of views and opinions on the benefits of cancer initiatives. National initiatives did not appear to wholly resolve issues in managing risk for all practitioners. Staff were concerned about the number of guidelines and priorities

For peer review only

they were expected to implement. These issues need to be considered by policymakers when developing and implementing new initiatives.

ARTICLE SUMMARY

Strengths and limitations of this study

- The study investigates the experiences of a range of primary care staff around implementing initiatives for the earlier diagnosis of cancer.
- The underlying concern in primary care is related to managing risk.
- The sample included a mix of practices with different practice characteristics and included those known to be engaged in national and regional initiatives and those who were less engaged.
- The sample was drawn from a single English region.

For peer review only

For peer review only - http://bmjopen.bmj.com/site/about/guidelines.xhtml

INTRODUCTION

Studies in the 1990s showed that UK cancer survival rates were worse than many other European countries, following which there has been two decades of concerted effort to expedite access to proven effective cancer treatments.[1] While there have been improvements in cancer survival latterly, the UK still lags behind ~~many~~ other countries with similar health care systems, which may be partly due to later stage of disease at presentation.[2]

Both nationally and internationally, primary care is seen to have a key role in improving cancer survival by reducing delays in diagnosis through promoting earlier presentation and through earlier detection and referral of those with symptoms for further specialist assessment.[3, 4] In the UK, national campaigns extol those with symptoms to see their general practitioner earlier; there are national referral guidelines for suspected cancer and a national system for urgent referral from primary to secondary care (two-week waiting-time initiative).[5] While these appear to have contributed to improved survival~~However~~, evidence also suggests that there may be further room for improvement:[6] there is practice variation in the use of the 'two-week' initiative and some patients are seen several times in primary care before referral.[7-9] A significant proportion of patients also present through emergency routes and have poorer survival.[10]

To further support primary care in the UK, resources were developed and/or disseminated by the Department of Health's National Awareness and Early Diagnosis Initiative (NAEDI), established in 2008, and the Royal College of General Practitioners (RCGP) including: audit and significant events analysis tools,[7, 8] general practitioner (GP) level cancer profile data,

For peer review only - http://bmjopen.bmj.com/site/about/guidelines.xhtml

safety netting recommendations and risk assessment tools.[11] Key initiatives have mainly focused on GPs, but other members of the primary care team may have key roles: an area which has been largely unexplored.

A number of international studies have been undertaken in attempts to understand the reasons behind delay in cancer diagnosis. Qualitative studies have mostly focussed on patient perspectives.[12-14] Only a few have explored primary care experiences and these have been mainly limited to decision-making processes or referral pathways [11, 15, 16] and from the perspective of the general practitioner.-[3, 11, 15, 16] In order to understand how a range of initiatives across the patient pathway in primary care could be more effective and the role of other members of the practice team, we explored the experiences of a range of primary care staff in supporting earlier presentation, detection and referral of those with symptoms suggestive of cancer. The study was undertaken in one region of England, which at that time was covered by the Lancashire and South Cumbria Cancer Network (LSCCN).

METHODS

This was a qualitative descriptive study utilising both individual and group-based interviews. It aimed to recruit staff from six practices who were differentially engaged with the national awareness and early diagnosis of cancer agenda. GP practices within LSSCN were stratified into one of three groups at the end of June 2012. High engagers had participated in at least one of the following: RCGP cancer audit, attendance at a course on early diagnosis or face-to-face meetings to discuss GP cancer profiles and action planning. Medium engagers had attended at least one regional meeting about cancer awareness. Low engagers were not

For peer review only

known to have engaged in any initiatives. Of 254 practices in the geographical area, there were 51 high engagers, 69 medium engagers and 134 low engagers.

Within each group, a random sample of 10 practices were sent a letter about the study, followed up with a phone call from the research team one week later. The initial aim was to recruit at least two practices in each stratum. Due to low uptake within the medium and low engager strata, these categories were merged and a random sample of a further 15 practices sent letters. Practices were offered a choice of either group or individual interviews as it was recognised that time constraints prevent some practice staff from taking part in group interviews and some may feel uncomfortable discussing the issues with colleagues. Five practices agreed to a group interview; with one group interview ~~was~~ undertaken in each practice.~~I~~ Individual interviews were the preferred method in only one practice: with four interviews ~~were~~ undertaken in this setting~~practice~~.

~~. Interviews (group and individual) were held on only one occasion in each practice.~~

~~Six practices agreed to take part in~~The semi-structured, audio-recorded interviews, which were attended by two researchers, occurred between September and October 2012. We recruited three practices in the high stratum and three practices in the merged medium and low stratum (a mix of low and medium engagers). The topic guide is outlined in Box 1. Thirty-nine participants took part in ~~group (n=5) or individual (n=4)~~the study; 35 took part in one of the five group interviews and four took part in individual interviews. Job roles included GP (n=9), receptionist (n=7), nurse (n=6), manager (n=6), secretary (n=5), health care assistant (n=3), medical student (n=2) and phlebotomist (n=1). ~~Within~~ In each practice, GPs, other clinical staff and administrative staff were involved in the interview~~eds~~. Practice characteristics are shown in table 1.

For peer review only

Table 1: Practice and interview characteristics

Practice characteristic	No. of practices
No of partners	
4 or more	3
3 or less	3
Deprivation Quintile*	
5 or 4 (more deprived)	3
2 or 3	3
1 (most affluent)	0
Population Density**	
<15%	2
15 to 39%	2
>=40%	2

*based on index of multiple deprivation of practice location 2010 (source: Department for Communities and Local Government, Indices of Deprivation 2010)
** Person per hectare based on practice location (source: ONS Neighbourhood Statistics)

Further detail at practice level is not presented Ddue to the level of confidentiality agreed with our participants.

we are unable to present further detail at practice level.

The interviews took between 38 and 67 minutes to complete, and between four and eleven staff took part in each practice. All transcribed data were entered into NVivo 10 and analysed using a Thematic Network Analysis approach.[17] This involved an iterative and cyclical process of reading and analysis to identify basic, organising and global themes within the dataset. Analysis was undertaken by two authors (GT and NC) independently on six transcripts initially, followed by an in-depth discussion and consensual validation of key themes. A further cycle of independent and collaborative analysis was then undertaken on a further sub-set of transcripts to ensure rigour and authenticity of the themes generated. All thematic decisions were discussed with the third author (PD). The study was approved by the

For peer review only - http://bmjopen.bmj.com/site/about/guidelines.xhtml

University of Central Lancashire 'STEMH' ethics committee and individual written informed consent was obtained from all participants.

RESULTS

Overall, practice staff were well aware of the two-week waiting-time initiative and had good knowledge about the national 'Be Clear on Cancer' cancer awareness public campaigns, but had less awareness about other initiatives specifically targeted at primary care. The key global theme to emerge from the interviews related to 'managing risk' within primary care:

> *"It's quite a tricky, nebulous area* […]. *The nature of general practice is that we're dealing every day with uncertainty…"* (Interview 6, Participant 3)

A key concern of staff was that policymakers and those implementing cancer initiatives did not fully understand how risk was managed within primary care. Cancer was only one priority and there was an abundance of initiatives for a variety of conditions which primary care staff were expected to implement. Three underpinning organising themes (and associated basic themes) of 'complexity', 'continuity' and 'conflict' highlighted the tensions and difficulties that primary care face in managing the risk of early detection and referral for cancer symptoms whilst dealing with complex symptoms and care systems, patient-led factors and target-focused care. An overview of the organising and basic themes is presented in figure 1. These themes are described and discussed, contextualised by participant quotes below.

Complexity

For peer review only - http://bmjopen.bmj.com/site/about/guidelines.xhtml

This theme highlighted the complexity of managing risk in early cancer diagnosis because of external factors including cancer symptom differentiation and the restrictions imposed by referral criteria; the multitude of services and professionals involved in diagnostic and assessment services and the plethora of policies and initiatives targeted at primary care practice.

Cancer disease and symptoms: Cancer diagnosis was an important priority area in primary care but diagnosis presented complex challenges. Cancer was a rare diagnosis in primary care although symptoms associated with cancer were common.

> *"We get lots of sore throats, and yet we get one tonsillar cancer every three* [years] *so sorting out the wheat from the chaff is a real challenge."* (Interview 3, Participant 3)

These complexities of diagnosis were compounded by what were considered rigid referral criteria, based on disease prevalence amongst those with symptoms, which led to cases being '*bounced back'* if they failed to meet diagnostic criteria. In attempts to manage what they considered was a risk to patients, some participants said, on occasion, they had to '*fudge'*, '*embellish'* or '*bend-the-rules'* to ensure assessment of patients for whom they had concerns:

> *"Even though it says on the form, "don't fill out this form unless they tick any boxes" but you find a box to tick, and usually for very good reason. And I think you'd only go, slightly over egging the presentation if you were pretty sure there was something there, something going on."* (Interview 4, Participant 4)

For peer review only - http://bmjopen.bmj.com/site/about/guidelines.xhtml

Fragmentation and access to diagnostic services: Primary care staff expressed difficulties accessing diagnostic services due to on-going service re-configurations and the involvement of multiple agencies. This led to fragmentation in terms of staff not always knowing who, or to which services, referrals could be made.

There were also some concerns about fragmented relationships between primary and secondary care with several participants feeling frustrated by restricted access to diagnostics for certain conditions. Some participants considered this was due to *'empire building'* by professionals justifying and *'preserving'* their service by retaining ownership of who was qualified to make referrals:

> *"I can send people for a CT scan if I have a concern about them, for some conditions, but not others. Well why not? I'm the one who's initiating the referral in the first place. You trust me to initiate the referral to pick the patient and prepare them so that you can come along and just arrange the scan and look at it."* (Interview 4, Participant 3)

Whilst some GPs wanted more direct access to diagnostics, this was not universal, and others highlighted the need for *'training'* prior to referrals being made.

Guidelines content and information overload: Participants referred to the usefulness of guidelines to help symptom differentiation and manage risk. Knowledge was felt to be experientially derived and referral guidelines only considered useful for less experienced doctors. Risk assessment tools were occasionally considered unnecessarily complex when

For peer review only - http://bmjopen.bmj.com/site/about/guidelines.xhtml

patients had 'red flag' symptoms and, as previously mentioned, sometimes restrictive for use in primary care:

> *"I mean, you see, from a GPs point of view, if somebody comes in who is fifty-five years of age, and passes blood in his stool, you need to exclude a cancer. Now, I don't want to know how many percentage of those people pass blood in the motion will have cancer, or how many will have piles, or inflammatory bowel disease or what have you."* (Interview 4, Participant 3)

Concerns were expressed about the number of guidelines available for different conditions and inconsistencies between different sources:

> *"And that's the problem 'cause last year there was a big campaign, "if you have a cough for more than three weeks you need to go and see a doctor", but NICE guidelines is six weeks.*" (Interview 6, Participant 4)

Participants described how the *'tsunami'* of new guidelines, care plans and initiatives meant it was difficult to keep up-to-date:

> *"And I thought 'that's probably a squamous cell carcinoma. That needs a two-week wait referral'. Then, I actually checked the guidelines, the guidelines say that "it's got to be over one centimetre and you've got to wait more than eight weeks really for it to grow" etcetera, etcetera* […] *and those things will change on a regular basis. Now, how do you expect an entire network of primary care physicians to stay current with all those guidelines and apply them religiously?"* (Interview 2, Participant 3).

For peer review only - http://bmjopen.bmj.com/site/about/guidelines.xhtml

Continuity

Practice staff expressed concern that a lack of continuity increased the risk of missing diagnoses and/or supporting the patient through a difficult period. This was highlighted through discussions about relationships between patients and clinicians; delays in information sharing across the primary and secondary care interface and patient follow-up after initial consultation.

Patient-practice relationships: Continuity-of-carer could not always be achieved in practice, even though it was felt that patients preferred it and its absence was thought to possibly contribute to diagnostic delay:

> *"People have been hopping around from one clinician to another and that, and you don't see the evolution of the story until it hits you in the face. Patients book on the day, they don't necessarily get in the person they normally see, they get in with the available, so that can disjoint things."* (Interview 3, Participant 4)

Staff felt that smaller, rural practices had closer relationships with their patients and their community, which would lead to earlier consultation. However, others felt awareness of cancer deaths was heightened in close-knit communities, which could reinforce negative views towards cancer and cancer survivorship amongst the local population and delay access to services.

For peer review only - http://bmjopen.bmj.com/site/about/guidelines.xhtml

Primary care and secondary care interface: Expedited assessment following GP referral of patients through the two-week wait initiative was perceived to work well by practice staff, but delays further on in the assessment process, and outside of primary care control, were felt to put patients at risk again and exacerbated their concerns:

> *"And, well, it's very difficult from our point of view, 'cause we're saying, "look, you know this may be nothing serious, however, I want to refer you on the system, you'll get an appointment in two weeks", and they do get seen, but then there's a massive delay, so, you know, then they're kinda all heightened up because they're thinking, "{whispered} the doctor thinks I've got cancer" and then nothing happens for ages, and it's really hard for the patients."* (Interview 3, Participant 5)

Practice-based follow-up: Patients themselves were also considered to cause delays if they failed to attend. Practices debated the extent to which they should chase these patients up and the risks if they did not:

> *"In the back of their mind they know they should, but if they face the fact that they're going back, are they are going to be faced with something else? You also have to be responsible for the fact that if that doesn't stop, you must persist, and accept the fact that, if you don't, then you could, somewhere down the line end up with something so serious that it's only going to be palliative."* (Interview 2, Participant 1)

Conflict

For peer review only

For peer review only - http://bmjopen.bmj.com/site/about/guidelines.xhtml

Within this theme, managing risk was related to primary care focus on targets; conflicting opinions about the role of non-clinical staff and about the worries and tensions generated by cancer awareness campaigns.

Prevention versus target driven care: ~~It is recognised that primary care can make a significant contribution to public health through promotion, in the practice population, of healthier lifestyles and earlier presentation of symptoms of diseases, where early intervention reduces morbidity and mortality, such as cancer.~~ Primary care staff considered cancer a public health priority but some felt that there was a tension between adopting preventative approaches in the practice such as promoting cancer awareness and early presentation and the way that primary care is currently funded through achieving specific targets mainly relating to the management of long-term conditions (Quality and Outcomes Framework):

> *"We're not as good with public health, with local public health initiatives, as maybe we should be necessarily. It's just time to do things, and those things aren't required of our core business, and when you have a very detailed contract that tells you what you will be paid for doing...."* (Interview 2, Participant 3)

Staff roles: There were conflicting views around the role of other practice staff in managing the risk of cancer detection and awareness, in particular with regard to reception staff. Some staff viewed the reception role as a health advisor, whereas others perceived their role to be purely administrative. Additionally, there were reservations about the ability of reception staff to relay cancer messages and how the public might feel about receiving health information from them:

For peer review only - http://bmjopen.bmj.com/site/about/guidelines.xhtml

> *"You're passing clinical responsibility to people who are non-clinical. It's alright for patients to speak to them, and that's fine, but, I'm not gonna use the receptionists as a source of information* […]. *I think anything clinical should be passed to the doctor full stop. I don't think there is any other role for the receptionist."* (Interview 4, Participant 3)

There were also concerns about potential litigation issues if reception staff were to give advice and how this could impact on the practice:

> *"I think it's also worth mentioning at this point, because of our roles, how they are, we get a lot of patients not happy about us supplying information, because we're not allowed to diagnose, obviously for obvious reasons, but if you was to imply something, that could come back on us twice as hard because you'd implied something that could be wrong, and therefore it is now your fault."* (Interview 1, Participant 1)

A further issue associated with staff roles in helping to manage risk was the identified benefits of having a chain of communication between all practice members. For example, receptionists being able to *'raise issues'* with the doctor if they know someone is coming in who has symptoms the doctor should be aware of but the patient *'may not say anything'*. This can also work the other way:

> *"Patients that I worry about, I will leave messages. I'll mention to reception or mention to people that I want to follow them up."* (Interview 6, Participant 3)

For peer review only

Others felt that the focus on the long-term conditions might provide opportunities for practice nurses to be more involved as they were often seeing patients who might be at higher risk of cancer because of their age, disease condition or lifestyle behaviours:

> *"Chronic disease, yeah, so diabetic, high blood pressure, chronic kidney disease, asthma, most patients will come and see the practice nurses for routine bloods, blood pressure, weight, everything like that. So often, GPs don't always see them, I mean they do the medication reviews, but, we flag to them anything that we're worried about."* (Interview 2, Participant 4)

Some could also see a role for members of the wider practice team:

> *"We'd say 'well who are the district nurses already going out to?' You know, you can work with your local pharmacists."* (Interview 2, Participant 2)

Cancer awareness: Although cancer fear was commonly acknowledged, staff were divided as to whether patients were afraid to consult the GP with cancer symptoms. Some felt that unhealthy lifestyle choices contributed to patient reluctance to present with cancer symptoms:

> *"The cancers where they feel that they may have contributed to it, like smoking, they tend to ignore because they don't want to be told that it may be their fault in a certain way, and they don't want to give up the lifestyle."* (Interview 6, Participant 4)

Cancer awareness campaigns were felt to be important, even though those who presented to the practice following the campaign were more likely to be those at least risk:

For peer review only - http://bmjopen.bmj.com/site/about/guidelines.xhtml

"It's the people who never come to the doctors that you want to hit, not the people that were already coming in anyway, and they tend to be ones that see those adverts. But then if you can get one person who wouldn't normally come in and you catch them, then it's better." (Interview 5, Participant 1)

Campaigns were also felt to create extra work for all practice members. There were complaints that cancer awareness campaigns tend to run without consideration of other local or national campaigns which may be running simultaneously which increased the risk that practice capacity to safely respond to patients was compromised:

"It's like it's alright saying putting in place to get them in, but it's the sheer volume, *isn't it? You can only cope with so much can't you?"* (Interview 5, Participant 1)

DISCUSSION

This study provides insights into the experiences of primary care staff who manage patients with symptoms ~~suggestive of~~associated with cancer. The overarching theme thato emerged was the need to manage risk so that cancer patients had a timely diagnosis and were assessed appropriately. The associated subthemes of complexity, continuity and conflict highlighted the tensions and difficulties faced by staff when attempting to manage these risks in modern practice.

For peer review only - http://bmjopen.bmj.com/site/about/guidelines.xhtml

The study was small, descriptive and exploratory. However, it covered a diverse geographical area and included practices with varying levels of engagement with awareness and early diagnosis initiatives. We undertook stratified sampling of the practices ~~based on the level of on engagement~~ to ensure we had a balance of perspectives so as to better inform policymakers of the possible barriers and drivers to the uptake of initiatives. The stratification was based on known engagement with a~~the~~ range of national and regional initiatives to promote earlier presentation, detection and referral of patients with symptoms suggestive of cancer available to the practices at that time.

The findings of our study suggest that national initiatives did not appear to wholly resolve issues ~~in~~ around managing risk for all practitioners. Rather, in some cases, these initiatives were felt to introduce inherent risks which staff had to find ways to overcome. ~~e~~For example, national cancer awareness campaigns ~~This included cancer awareness campaigns . However, these campaignswhich, while felt~~ were felt to be very important to encourage patients who would otherwise delay diagnosis. However, as these ~~also~~ campaigns appeared to increase consultation rates from those at lesser risk,~~;~~ this was perceived to place~~d . This placed~~ additional burden on the practice.

There ~~were~~ were also concerns amongst our participants about practice-based initiatives ~~introduced to directly support practices~~. The International Cancer Benchmarking Partnership ~~has~~ observed lower survival rates in Denmark and the UK compared to other countries in their study, ~~which has~~ and raised concerns about the role of primary care gatekeeping in delayed diagnosis.-[2] -This observation appears to be supported by a wider study of European countries which also demonstrated lower cancer survival in those with primary care based gatekeeper systems.[18] Primary care gatekeeping may reduce the burden on specialist

For peer review only

For peer review only - http://bmjopen.bmj.com/site/about/guidelines.xhtml

care but may also contribute to diagnostic delay through the restriction of access to these services. In the UK, national referral guidelines and the two-week waiting-time initiative should help alleviate this problem for those with symptoms associated with cancer.

Participants in our study considered ~~highlighted~~that timely diagnosis was as an essential part of their role in cancer care. ~~This~~ An Australian study had a similar finding ~~was A finding reported in an Australian study~~,.[16] , but highlighted that ~~who This study also identified that the~~the resources spent gaining timely access to specialist opinion were a major issue for their practitioners. In our study this was universally ~~appeared to be~~ considered less of a problem because of the two-week waiting-time initiative, ~~. This initiative was universally embraced by participants as it was felt to work well in facilitating timely assessment. However,~~ but ~~there were still~~ concerns were expressed that lack of direct access to diagnostic investigations and poor communication between primary and secondary care put patients at risk ~~due to~~ of extended delays~~,~~. Similar concerns were ~~as~~ reported in a study undertaken in Ireland.[3] Some participants in our study felt that the referral criteria for the two-week waiting-time initiative were too restrictive. This ~~might.~~ ~~and this~~ le~~a~~d to practitioners, on occasion, subverting the referral system to ensure that patients they considered were at risk, but who did not fit the referral criteria, could be assessed in a timely manner. Such concerns are not unfounded; one study has shown that 8% of patients felt by GPs to have cancer, but who did not have symptoms which fit referral criteria for cancer, were subsequently diagnosed with cancer ~~cancer patients do not have symptoms which fit referral criteria~~.[1~~8~~9] This may be because the presenting signs and symptoms had a lower predictive value for cancer than those included in the guidelines. Nevertheless, there is high compliance with guidelines and some limited evidence that referral guidelines contribute, in part, to diagnostic delay reduction.[6, 1~~8~~9]

For peer review only

For peer review only - http://bmjopen.bmj.com/site/about/guidelines.xhtml

Diagnostic errors leading to primary care malpractice claims are common.[~~19~~20] Researchers suggest that although GPs are more likely to correctly diagnose patients with cancer than miss cases, there are a disproportionate number of deaths amongst the latter.[2~~0~~1,~~-~~22~~21~~] Diagnostic complexity is compounded by the frequency of consultations for symptoms associated with cancer,~~-~~[2~~1~~2, 2~~2~~3] a fear highlighted by practitioners in this study. A study in Norway, which followed up patients presenting with cancer warning signs to their GP, suggests that patients with cancer may be missed if multiple warning signs and symptoms are not considered.[2~~1~~2] Retrospective studies of practice-based data have identified combinations of warning signs and symptoms which may be associated with increasing~~ the~~ likelihood of cancer.[2~~3~~4-2~~4~~5] These have been used to inform risk assessment tools to aid decision-making, with algorithms and probabilities of risk based on demographic characteristics, lifestyle factors, symptoms and/or attendance frequency.[11] Some have been disseminated nationally, and there is evidence from both quantitative and qualitative studies that they affect GPs' decisions to refer.[11] However, in our study, some participants had similar concerns about these tools as they did about guidelines. A recent analysis of significant event audits in lung cancer highlighted how cancer can mimic other diagnoses and have atypical presentation.[7] Guidelines and risk assessment tools are analytical tools, and although statistical probabilities are based on uncertainty, some participants in our study felt ~~they may be perceived to~~ they may introduce a level of certainty which fails to encapsulate practitioners' tacit concerns about patients. Previous work in primary care decision-making has emphasised the analytical over the experiential, but recent research , including in cancer diagnosis, suggests that experiential knowledge may have a role and may be more responsive to ~~individual~~ the patient as a person.[15, 2~~5~~6-2~~8~~7] Others have found that better diagnostic decisions about urgent referrals appear to be made by older doctors ~~-~~[2~~2~~3] and, as commented

For peer review only

For peer review only - http://bmjopen.bmj.com/site/about/guidelines.xhtml

on by some of our participants, that guidelines and risk assessment tools may be more useful for newer practitioners who have yet to develop problem solving strategies.[2~~8~~9]

It has also been observed that, unlike in acute care, decision-making in primary care partly involves an understanding of the patient context and perspective.[~~29~~30] Our study participants highlighted concerns that lack of continuity of carer may lead to diagnostic delays. Relational continuity was felt to be a particular problem in larger practices and urban settings. Other studies~~s~~ suggest that fragmentation of primary care and shorter consultation times has led to a lack of continuity-of-care or carer, which may hinder early diagnosis as cancer presentation can be complex in those with ~~multiple~~ co-morbidities.[16, 3~~0~~1, 3~~1~~2] In a qualitative study of lung cancer patients in New Zealand, patients felt that not always seeing the same GP could lead to delays in diagnosis due to poor follow up.[3~~2~~3] In a study in Denmark, the -authors suggest that perceived lack of accessibility and ~~the~~ doctor-patient relationship were associated with patient delay in seeking advice about cancer symptoms.[3~~3~~4] Others have found that confidence and trust in a doctor were more important predictors of cancer detection than ease of access and choice of preferred doctor.[3~~4~~5]

In our study, participants highlighted how other practice team members, such as nurses who are in more regular contact with patients with long-term conditions, could raise symptom awareness or be more alert to ~~changes in, or new,~~ symptoms suggestive of cancer. In the US and Canada, patient navigators are being used to support patients through the complex systems found in cancer management and care.~~-~~[36] It has also been suggested that these roles could be extended to support patients during the diagnostic, referral and assessment processes and ensure appropriate follow up of investigati~~ve results~~ons.[3~~5~~7] In our study,

For peer review only

some staff felt that receptionists could act as navigators in terms of directing patients with symptoms to see the GP.~~[36]~~ Low levels of knowledge about some cancer symptoms has been demonstrated in non-medical staff.[3~~7~~8] However, our study highlighted that professional boundaries and concerns about litigation~~ which~~ might ~~also ~~impede such initiatives.~~;~~ D~~d~~isclosures or discussions of diagnostic issues within a public reception location also raised ethical concerns. Research within primary care suggests that facilitation of communities of practice and interdisciplinary knowledge sharing may help to identify the role of other practice team members in promoting earlier cancer presentation and diagnosis;[39~~8~~] although training needs should be addressed.[38~~7~~]

This study has highlighted that a 'one-size-fits-all' approach to implementing initiatives is unlikely to succeed as practitioners expressed a range of views and opinions on the benefits of different initiatives. Lack of implementation of initiatives in primary care is not always because of resistance to the initiatives themselves, but sometimes because of the sheer number across a range of priority areas that practices are expected to implement, often simultaneously. Policymakers should consider more carefully how these impact on primary care, how they can be embedded into practice systems and emphasise and exploit synergies with other disease conditions.

For there to be greater success of initiatives aimed at promoting earlier presentation, detection and referral in primary care, there needs to be further work on understanding how primary care manage risk in the face of inherent uncertainty, organisational changes and competing priorities.

For peer review only - http://bmjopen.bmj.com/site/about/guidelines.xhtml

ACKNOWLEDGEMENTS: Our thanks to the practices who participated in this study and to the staff of the Lancashire and South Cumbria Cancer Network who assisted us.

COMPETING INTERESTS: PD was public health lead for LSCCN and NC is part-funded by a grant from LSCCN; PD was also funded by LSCCN to undertake evaluative work.

FUNDING: This work was supported by Lancashire and South Cumbria Cancer Network (LSCCN) as part of the National Cancer Awareness and Early Detection initiative (NAEDI) funding 'cancer networks supporting primary care'.

CONTRIBUTIONS:

NC recruited participants, carried out interviews, transcribed the interviews, coded the transcripts, contributed to the development of the coding framework, and contributed to the writing of the manuscript.

GT carried out interviews, coded the transcripts, contributed to the development of the coding framework and contributed to the writing of the manuscript.

PD designed the study, collated practice engagement data, contributed to the development of the coding of the framework and contributed to the writing of the manuscript.

DATA SHARING: No further data are available

REFERENCES

1. Haward R. The Calman-Hine report: a personal retrospective on the UK's first comprehensive policy on cancer services. *The Lancet Oncology*. 2006;7(4):336-46.

For peer review only

For peer review only - http://bmjopen.bmj.com/site/about/guidelines.xhtml

2. Coleman MP, Foreman D, Bryant H, et al. Cancer survival in Australia, Canada, Denmark, Norway, Sweden, and the UK, 1995—2007 (the International Cancer Benchmarking Partnership): an analysis of population-based cancer registry data. *The Lancet*. 2011;377:127–38. doi:10.1016/S0140-6736(10)62231-3.
3. Daly H, Collins C. Barriers to early diagnosis of cancer in primary care: a needs assessment of GPs. *Ir Med J*. 2007; 100(10):624-26
4. Ingebrigsten SG, Scheel BI, Hart B, et al. Frequency of 'warning signs of cancer' in Norwegian general practice, with prospective recording of subsequent cancer. *Fam Pract*. 2013;30:153-160
5. National Institute for Clinical Excellence. Clinical Guideline 27. Referral guidelines for suspected cancer. 2005. http://www.nice.org.uk/nicemedia/live/10968/29814/29814.pdf accessed 3/1/2014.
6. Neal RD, Din NU, Hamilton W, et al. Comparison of cancer diagnostic intervals before and after implementation of NICE guidelines: analysis of data from the UK General Practice Research Database. *Br J Cancer*. 2014;110(3):584-92. doi:10.1038/bjc.2013.791. 4.
7. Mitchell ED, Rubin G, MacLeod U. Understanding diagnosis of lung cancer in primary care: qualitative synthesis of significant event audit reports. *Br J Gen Pract*. 2013;63(606):e37-46. doi:10.3399/bjgp13X660760.
8. Royal College of General Practitioners. Clinical Innovation and Research Centre. National Audit of Cancer Diagnosis in Primary Care. 2011. http://www.rcgp.org.uk/news/2011/november/~/media/Files/News/National_Audit_of_Cancer_Diagnosis_in_Primary-Care.ashx. accessed 03/01/2014.

Field Code Changed

For peer review only - http://bmjopen.bmj.com/site/about/guidelines.xhtml

9. Meechan D, Gildea C, Hollingworth L, et al. Variation in use of the 2-week referral pathway for suspected cancer: A cross-sectional analysis. *Br J Gen Pract*. 2012; 62(602):e590–97. doi:10.3399/bjgp12X654551.
10. Elliss-Brookes L, McPhail S, Ives A, et al. Routes to diagnosis for cancer - determining the patient journey using multiple routine data sets. *Br J Cancer*. 2012;107(8):1220-26. doi:10.1038/bjc.2012.408.
11. Hamilton W, Green T, Martins T et al. Evaluation of risk assessment tools for suspected cancer in general practice: a cohort study. *Br J Gen Pract*. 2013;63(606):e30-36. doi:10.3399/bjgp13X660751.
12. Emery JD, Walter FM, Gray V, et al. Diagnosing cancer in the bush: a mixed-methods study of symptom appraisal and help-seeking behaviour in people with cancer from rural Western Australia. *Fam Pract*. 2013 Jun;30(3):294-301.doi: 10.1093/fampra/cms087
13. Pedersen AF, Hansen RP, Vedsted P. Patient delay in colorectal cancer patients: associations with rectal bleeding and thoughts about cancer. *PLoS One*. 2013:Jul 22;8(7):e69700.doi: 10.1371/journal.pone.0069700
14. Marlow LA, McGregor LM, Nazroo JY, et al. Facilitators and barriers to help-seeking for breast and cervical cancer symptoms: a qualitative study with an ethnically diverse sample in London. *Psychooncology*. 2013;Dec 19.doi:10.1002/pon.3464
15. Johansen ML, Holtedahl KA, Rudebeck CE. How does the thought of cancer arise in a general practice consultation? Interviews with GPs. *Scand J Prim Health Care*. 2012;30(3):135-40. doi:10.3109/02813432.2012.688701.
16. Mitchell GK, Burridge LH, Colquist SP, et al. General Practitioners' perceptions of their role in cancer care and factors which influence this role. *Health Soc Care Community.* 2012; 20(6):607-16. doi:10.1111/j.1365-2524.2012.01075.x

For peer review only - http://bmjopen.bmj.com/site/about/guidelines.xhtml

17. Attride-Stirling J. Thematic networks: an analytic tool for qualitative research. *Qualitative Research*. 2001;1(3):385-405. doi:10.1177/146879410100100307.

18. Vedsted P and Olesen F. Are the serious problems in cancer survival partly rooted in gatekeeper principles? An ecologic study. *Br J Gen Pract*. 2011;DOI:10.3399/bjgp11X588484.

19. Baughan P, Keatings J, O'Neill B. Urgent suspected cancer referrals from general practice: audit of compliance with guidelines and referral outcomes. *Br J Gen Pract*. 2011; doi:10.3399/bjgp11X606591.

20. Wallace E, Lowry J, Smith SM, et al. The epidemiology of malpractice claims in primary care: a systematic review. *BMJ Open*. 2013;3:e002929. doi:10.1136/bmjopen-2013-002929.

~~21. Balla J, Heneghan C, Thompson M, et al. Clinical decision making in a high-risk primary care environment: a qualitative study in the UK. *BMJ Open*. 2012;2:e000414. doi:10.1136/bmjopen-2011-000414~~

21. Wallis K, Dovey S. No fault compensation for treatment injury in New Zealand: identifying threats to patient safety in primary care. BMJ Quality Safety. 2011;20(7):587-91. doi: 10.1136/bmjqs.2010.0476906.

22. Scheel BI, Ingebrigsten SG, Thorsen T, et al. Cancer suspicion in general practice: the role of symptoms and patient characteristics, and their association with subsequent cancer. *Br J Gen Pract*. 2013;doi:10.3399/bjgp13X671614

23. Jiwa M, Gordon M, Arnet H, et al. Referring patients to specialists: a structured vignette survey of Australian and British GPs. *BMC Fam Pract*. 2008; 9:2.doi: 10.1186/1471-2296-9-2.

24. Emery J. Editorial: Assessment of cancer risk in men and women. *Br J Gen Pract*. 2013;63(606):4-5.doi:10.3399/bjgp13X660607.

Field Code Changed

For peer review only - http://bmjopen.bmj.com/site/about/guidelines.xhtml

25. Hamilton W. The CAPER studies: five case-control studies aimed at identifying and quantifying the risk of cancer in symptomatic primary care patients. *Br J Cancer*.2009;101: S80-S86 doi:10.1038/sj.bjc.6605396

26. André M, Andén A, Borgquist L, et al. GPs' decision-making-perceiving the patient as a person or a disease. *BMC Fam Pract*. 2012;13:38. doi:10.1186/1471-2296-13-38.

27. Stolper E, Van de Wiel M, Van Royen P, et al. Gut feelings as a third track in general practitioners' diagnostic reasoning. *J Gen Intern Med*. 2011;26(2):197-203. doi:10.1007/s11606-010-1524-5.

28. Zwolsman S, te Pas E, Hooft L, et al. Barriers to GPs' use of evidence-based medicine: a systematic review. *Br J Gen Pract*. 2012;62(600):e511-21. doi: 10.3399/bjgp12X652382.

29. Salah A, Hassan A. Cognitive schemes and strategies in diagnostic and therapeutic decision making: a primer for trainees. *Perspect Med Educ.* 2013; 2:321-31. doi:10.1007/s40037-013-0070-3

30. O'Riordan M, Dahinden A, Aktürk Z, et al. Dealing with uncertainty in general practice: an essential skill for the general practitioner. *Qual Prim Care*. 2011;19:175-81.

31. Brindle L, Pope C, Corner J, et al. Eliciting symptoms interpreted as normal by patients with early lung cancer. *BMJ Open*. 2012;2:e001977. doi:10.1136/bmjopen-2012-001977.

32. Round T, Steed L, Shankleman J, et al. Primary care delays in diagnosing cancer: what is causing them and what can we do about them? *J R Soc Med*. 2013;106(11):437-40. doi:10.1177/0141076813504744.

For peer review only - http://bmjopen.bmj.com/site/about/guidelines.xhtml

33. Walton L, McNeill R, Stevens W, et al. Patient perceptions of barriers to the early diagnosis of lung cancer and advice for health service improvement. *Fam Pract*. 2013;30:439-44 doi:10.1093/fampra/cmt001

34. Hansen RP, Vedsted P, Sokolowsk I, et al. General practitioner characteristics and delay in cancer diagnosis: a population-based cohort study. *BMC Fam Pract*. 2011; 12:100

35. Rogers S, Gildea C, Meechan D, et al. Access, continuity of care and consultation quality: which best predicts urgent cancer referrals from general practice? *J Public Health (Oxf).* 2014 Jan 23. [Epub ahead of print]

36. Dohan D, Schrag D. Using navigators to improve care of underserved patients. *Cancer*. 2005;104:848-55. doi:10.1002/cncr.21214.

37. Lee J-H, Fulp W, Wells KJ, et al. Effect of patient navigation on time to diagnostic resolution among patients with colorectal cancer-related abnormalities. *J Canc Educ.* 2014;29:144-50

38. Cook N, Hart A, Nuttall K, et al. A telephone survey of cancer awareness among frontline staff: informing training needs. *Br J Cancer*. 2011;105(3):340-45. doi:10.1038/bjc.

39. Kislov R, Walshe K, Harvey G. Managing boundaries in primary care service improvement: a developmental approach to communities of practice. *Implement Sci*. 2012;7:97. doi:10.1186/1748-5908-7-97.

For peer review only - http://bmjopen.bmj.com/site/about/guidelines.xhtml

Figure 1: organising themes of complexity, continuity and conflict with their associated basic themes, centred around the global theme of managing risk in primary care

For peer review only - http://bmjopen.bmj.com/site/about/guidelines.xhtml

Box 1: Semi-structured interview schedule

- Why do you think people with symptoms suggestive of cancer do not present, get seen or diagnosed or referred earlier?

- What do you know about the initiatives that concern the earlier presentation, diagnosis and referral of cancer symptoms?
    - Prompts around specific tools/initiatives/referral criteria

- Have you accessed any local training events concerning the identification, referral of cancer patients?
    - Prompts around specific training attended, and how learning is usually undertaken

- Overall, what is working well in terms of the implementation and use of these initiatives?

- Overall, have you experienced any/or what do you consider to be the main barriers in the implementation/access/use of these various initiatives?

- Are there any practice based issues that may affect the early identification and referral for cancer patients?
    - Prompts around staff and practice issues, communication, administration

- Overall, what do you think local practices could do to help promote and diagnose cancer symptoms?
    - Prompts around team roles and other practice issues

- Do you know about the Lancashire and South Cumbria Cancer Network and what their role is?
    - Prompts around extent of engagement, attitudes and areas for improvement

- What is your opinion concerning the forthcoming 'access to diagnostics' initiative (initiative for practices to make direct referrals to diagnostics such as x-rays, CT scans, ultrasound)?
    - Prompts around who should make the referral

- Are there any additional support mechanisms/external to the practice that need to be in place to help with promotion/diagnosis and referral?

- Any further issues or concerns you would like to raise about this work?

For peer review only - http://bmjopen.bmj.com/site/about/guidelines.xhtml

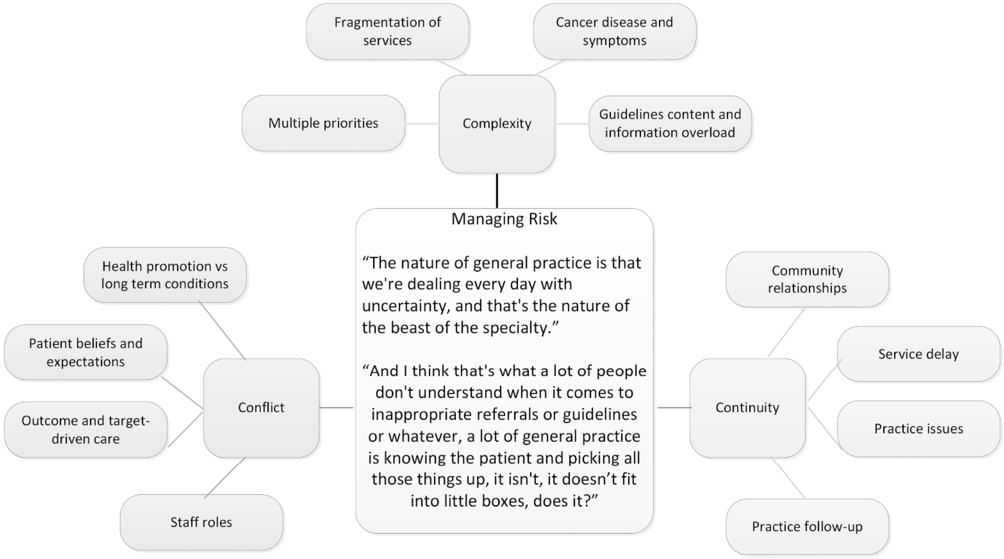

Figure 1: organising themes of complexity, continuity and conflict with their associated basic themes, centred around the global theme of managing risk in primary care
233x130mm (300 x 300 DPI)

For peer review only - http://bmjopen.bmj.com/site/about/guidelines.xhtml
